# Natural flavonoids in multiple sclerosis: molecular insights and emerging therapeutic strategies

**DOI:** 10.3389/fimmu.2026.1888423

**Published:** 2026-07-30

**Authors:** Abuyaseer Abusaliya, Mariam Al Shamsi, Hiba Orsud, Zakeya Al Rasbi, Sumaya Zoughbor, Nura Joher

**Affiliations:** 1 Department of Medical Microbiology and Immunology, College of Medicine and Health Sciences, United Arab Emirates University, Abu Dhabi, United Arab Emirates; 2 College of Dentistry, Ajman University, Ajman, United Arab Emirates

**Keywords:** flavonoids, multiple sclerosis, neuroinflammation, pharmacology, remyelination

## Abstract

Multiple sclerosis is a chronic immune mediated disease in which current disease modifying therapies reduce inflammatory relapses but incompletely address neurodegeneration and remyelination. Natural flavonoids are pleiotropic polyphenols that can modulate immune and glial signaling, oxidative stress, and mitochondrial function. This review synthesizes evidence from experimental models and human studies on flavonoids relevant to multiple sclerosis, emphasizing mechanisms involving NF-κB, Nrf2, inflammasome signaling, and microglia and macrophage polarization that shape oligodendrocyte precursor cell differentiation and remyelination permissiveness. We highlight structure activity features, metabolism and glycosylation that govern exposure, and discuss translational barriers including low and variable bioavailability, limited blood brain barrier penetration, standardization, and potential interactions with approved therapies. Emerging enabling strategies are reviewed, including lipid and polymeric nanocarriers, stimuli responsive delivery, systems biology and multi omics target discovery, network pharmacology for multi target prioritization, microbiome informed approaches, and synthetic biology for scalable production and derivative optimization. Overall, preclinical studies consistently support anti-inflammatory and neuroprotective effects, while clinical evidence remains early and mixed, underscoring the need for well powered trials with pharmacokinetic and pharmacodynamic endpoints.

## Graphical abstract

**Figure:**
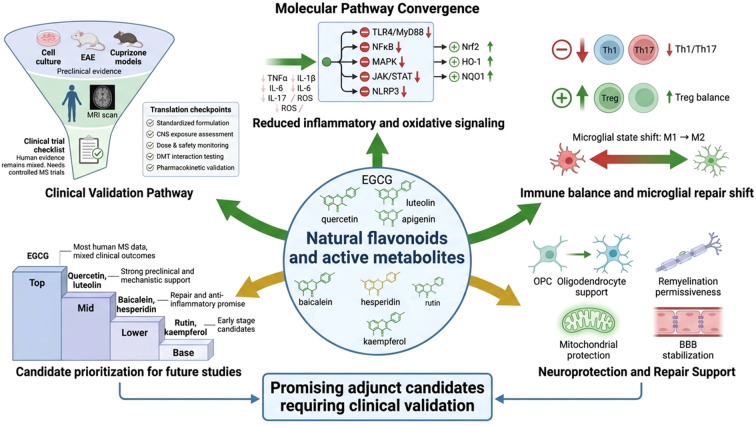

## 1 Introduction

Multiple sclerosis (MS) is one of the most complex and disabling neurological diseases of our time, with an unpredictable course and multiple symptoms that can affect millions around the world (1–3). This chronic autoimmune disease manifests through the immune system’s misguided attack on the central nervous system, leading to inflammation, demyelination, and subsequent neurological dysfunction (4). As medical science continues to grapple with the challenges of MS management, researchers have increasingly turned their attention to natural compounds that might offer novel therapeutic avenues. Among these, flavonoids, a diverse group of plant-derived polyphenolic compounds have emerged as particularly promising candidates for MS intervention (5–7). This structured narrative review summarizes current evidence on flavonoids in MS relevant experimental and human studies, with attention to mechanisms, translational barriers and future research needs.

## 2 Methodology

This review was prepared as a structured narrative review rather than a formal systematic review or meta-analysis. Literature searches were conducted from database inception to December 2025. Relevant literature was identified using PubMed, MEDLINE, Scopus, Web of Science, Google Scholar and ClinicalTrials.gov. Searches combined terms for multiple sclerosis and related models, including “multiple sclerosis”, “experimental autoimmune encephalomyelitis”, “EAE”, “cuprizone”, “demyelination” and “remyelination”, with terms for flavonoids and named compounds, including “flavonoid”, “flavonol”, “flavone”, “flavanone”, “isoflavone”, “anthocyanidin”, “flavanol”, “quercetin”, “EGCG”, “hesperidin”, “luteolin”, “rutin”, “baicalein”, “apigenin’, “kaempferol”, “genistein” and “naringenin”. Additional searches covered neuroinflammation, “NFκB”, “Nrf2”, “TLR MyD88”, “inflammasome”, “microglia”, “oligodendrocyte precursor cells”, “blood brain barrier”, “pharmacokinetics”, “metabolism”, “safety”, and “disease modifying therapy interactions”. We prioritized MS specific human studies, randomized controlled trials, EAE studies, cuprizone demyelination studies and relevant CNS, immune cell or blood brain barrier models. Studies from other neurological disease models or non-neurological systems were used only when they clarified mechanisms that could be relevant to MS, and these were described as indirect evidence. Because this article was designed as a structured narrative review, searches were iterative and topic driven rather than PRISMA based. Therefore, formal duplicate independent screening, a PRISMA flow diagram, and exact numerical screening counts were not generated. To improve transparency, studies were selected when they provided direct or mechanistic evidence relevant to flavonoids, flavonoid related compounds, MS, EAE, cuprizone demyelination, remyelination, neuroinflammation, oxidative stress, glial activation, blood brain barrier transport, pharmacokinetics, metabolism, safety, or interactions with disease modifying therapies.

Inclusion criteria were English language primary research or high-quality review articles reporting flavonoid or flavonoid related effects on MS, demyelination, neuroinflammation, oxidative stress, glial activation, remyelination, pharmacokinetics, metabolism, safety or blood brain barrier transport.

Exclusion criteria were studies without clear compound identity, studies with no mechanistic or translational relevance to MS, and non-MS studies used as direct evidence for clinical efficacy. Evidence was appraised narratively according to study type, MS specificity, model limitations, dose and route information, and outcome relevance.

## 3 Key players in MS onset and progression: the cellular and inflammatory landscape

### 3.1 Adaptive immune system: T and B cells in autoimmunity

The initiation of MS is primarily driven by autoreactive CD4^+^ T helper (Th) cells that recognize myelin-derived antigens presented by antigen-presenting cells (APCs) in peripheral lymphoid organs. The role of autoreactive CD4+ T cells mainly Th1 and Th17 cells have been intensively studied as major players in the disease pathogenesis. Th1 cells and their signatory proinflammatory cytokines, mainly interferon-gamma (IFN-γ) and tumor necrosis factor-alpha (TNF-α), were reported to contribute to the activation of the antigen presenting cells and promoting BBB disruption through upregulation of adhesion molecules and matrix metalloproteinases (MMPs) (8, 9). These actions facilitate the infiltration of autoreactive immune cells into the CNS parenchyma, initiating focal inflammatory lesions. Th17 cells are characterized by their production of interleukin-17 (IL-17), IL-21 and IL-22 and are critical in the early phase of disease by inducing neutrophil recruitment and disrupting the integrity of the BBB (10, 11). IL-17 activates astrocytes and endothelial cells to produce chemokines (e.g., CXCL1, CXCL8) and inflammatory mediators, increasing local inflammation. Th17 cells show a higher tropism for the central nervous system due to the expression of chemokine receptors such as CCR6 that directs them to CCL20-expressing cells in the choroid plexus and meninges (12).

Active MS lesions also harbor many autoreactive CD8^+^ cytotoxic T lymphocytes (CTLs) that directly contribute to tissue destruction, in addition to CD4^+^ T cells. These cells detect antigen presented by MHC class I molecules on ODCs and neurons, resulting in perforin- and granzyme-mediated lysis of target cells (13). Their existence is strongly correlated with axonal damage and disease progression, especially in progressive types of MS. On the contrary, regulatory T cells (Tregs) responsible for maintaining immunological tolerance by suppressing autoreactive T cells through IL-10, TGF-β and CTLA-4 are commonly reduced in numbers or functionally impaired in MS patients (14, 15). Deficit in immunoregulation leads to uncontrolled activation of pathogenic T-cell subsets, which contribute to persistent autoimmunity.

B cells are not simply classical antibody producing cells but also important participants in the pathogenesis of MS. They are antigen presentation cells (APCs) and are highly efficient in activating myelin-reactive T lymphocytes through MHC class II and co-stimulatory molecules (e.g. CD80/CD86) (16). B cells also release proinflammatory cytokines such as IL-6 and granulocyte-macrophage colony-stimulating factor (GM-CSF) that promote Th1 and Th17 development and activation of myeloid cells (17). Plasma cells (derived from B cells) create oligoclonal IgG bands in the CNS, which are characteristic of intrathecal antibody production, and seen in >95% of MS patients by study of cerebrospinal fluid (CSF) (18). These antibodies might play a role in complement-mediated demyelination and opsonization of myelin debris.

### 3.2 Innate immunity: microglia, macrophages and astrocytes

The innate immune system is a major player in the start and persistence of CNS inflammation in MS. In MS lesions, resident microglia and infiltrating monocyte-derived macrophages are the main phagocytic cells and show plasticity in their phenotype that governs their activity. In acute and active lesions these cells acquire a proinflammatory M1 phenotype with generation of reactive oxygen species (ROS), inducible nitric oxide synthase (iNOS), IL-1β, TNF-α and IL-12 (19). IFN-γ and Toll-like receptor (TLR) signaling generate M1 polarization that causes demyelination, oxidative stress, and neuronal damage. Sustained M1 stimulation causes lesion growth and remyelination deficit. On the other hand, M2-polarized microglia/macrophages secrete anti-inflammatory chemicals such as IL-10, TGF-β, arginase-1 and CD206 and participate in tissue healing, debris clearance and stimulation of remyelination (20). They support the development of oligodendrocyte precursor cells (OPCs) and produce growth factors such as IGF-1 and neurotrophins. M1/M2 balance is a key predictor of illness outcome and microglial reprogramming is a viable therapeutic target.

**Figure 1:**
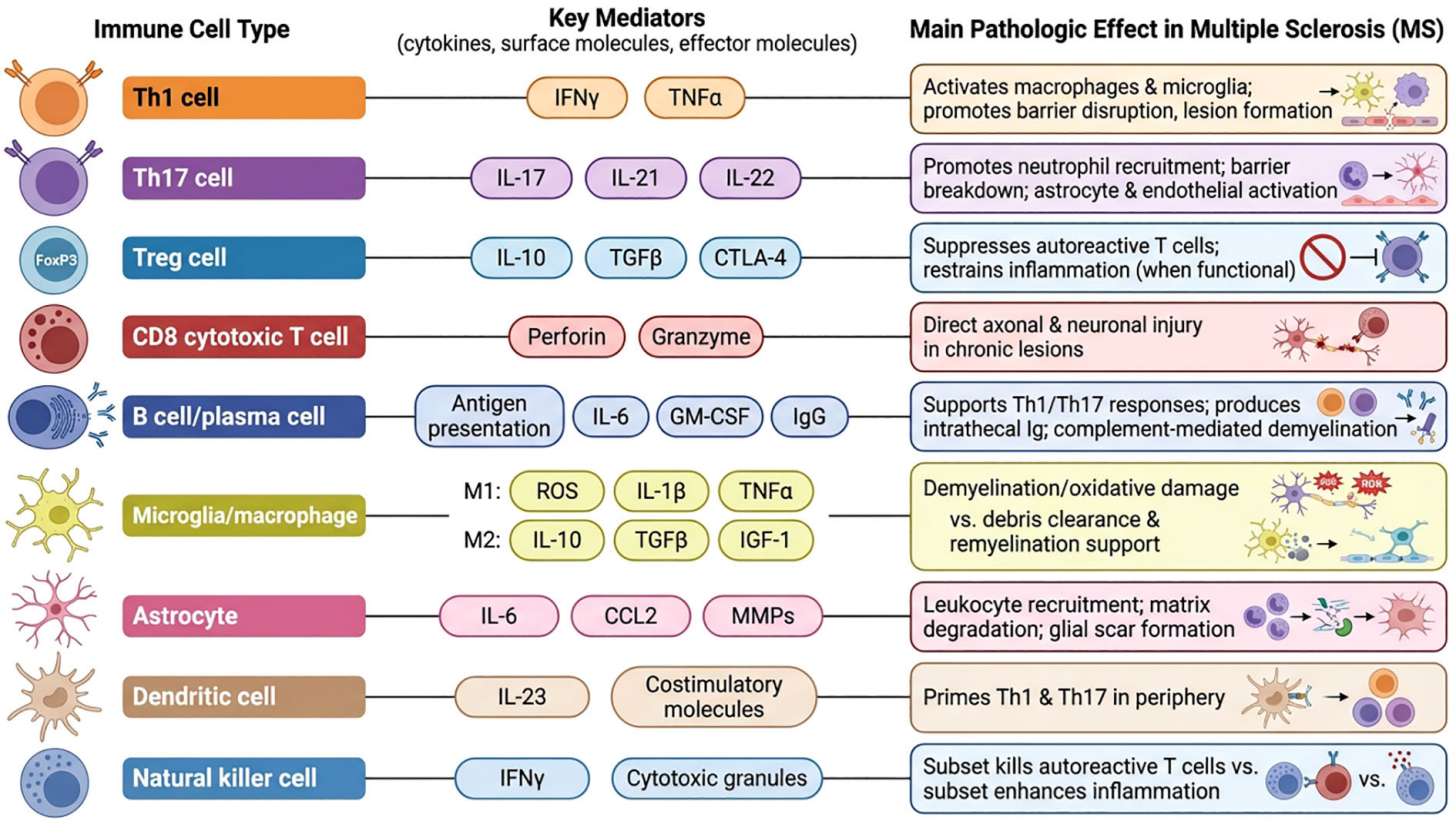
Key player immune cell and its signature cytokines and pathologic effect in MS disease progression.

Astrocytes were once assumed to be passive support cells, but are now known to actively influence neuroinflammation. They can be activated by cytokines (e.g., IL-1β, TNF-α) or danger signals to release a plethora of inflammatory mediators, including IL-6, CCL2 (MCP-1), and MMPs, that promote leukocyte recruitment and degradation of the extracellular matrix (21). In chronic MS, astrocytes are involved in the creation of glial scars, which constitute a physical and physiological barrier to axonal regeneration and remyelination (22). However, accumulating data supports neuroprotective roles of several astrocyte subsets, suggesting their functional heterogeneity. Other Immune Modulators are Dendritic Cells and Natural Killer Cells. Dendritic cells (DCs) are essential mediators of the interface between innate and adaptive immune responses. DCs can acquire antigens from the CNS (presumably translocated by meningeal lymphatic arteries) and transmit them to naive T cells in peripheral lymphoid organs to induce their priming and differentiation into pathogenic Th1/Th17 effectors (23). Additionally, DCs secrete IL-23, a cytokine involved in the maintenance of the Th17 phenotype. This suggests that NK cells have a dual role in MS. Some subsets (e.g., CD56^bright^) are regulatory, eliminating activated autoreactive T cells and DCs, while other subsets (e.g., CD56^dim^) can worsen inflammation through IFN-γ production (24). Altered NK cell function has been observed in MS patients, suggesting their involvement in immune dysregulation. The overall immune cells with their key modulators and its impact on MS were summarized in **Figure 1**.

### 3.3 Molecular signaling pathways in MS pathogenesis

At the molecular level, MS progression involves several interconnected signaling pathways regulating immune activation, glial responses, oxidative stress and tissue damage. The signaling pathways nuclear factor kappa B (NF κB), Janus kinase/signal transducer and activator of transcription (JAK/STAT), mitogen activated protein kinase (MAPK), phosphoinositide 3 kinase/protein kinase B/mammalian target of rapamycin (PI3K/Akt/mTOR), nuclear factor erythroid 2 related factor 2 (Nrf2) and inflammasome are involved in the production of cytokines, differentiation of Th1 and Th17 cells, activation of glia, oxidative injury and failure of remyelination. These pathways do not act independently but create an interacting network linking peripheral immune activation to CNS inflammation and neurodegeneration (25–29). Flavonoids can impact multiple points in this network, so their pathway specific actions are discussed together in the integrated flavonoid signaling section.

## 4 Classification and mechanisms of current DMTs

**Figure 2:**
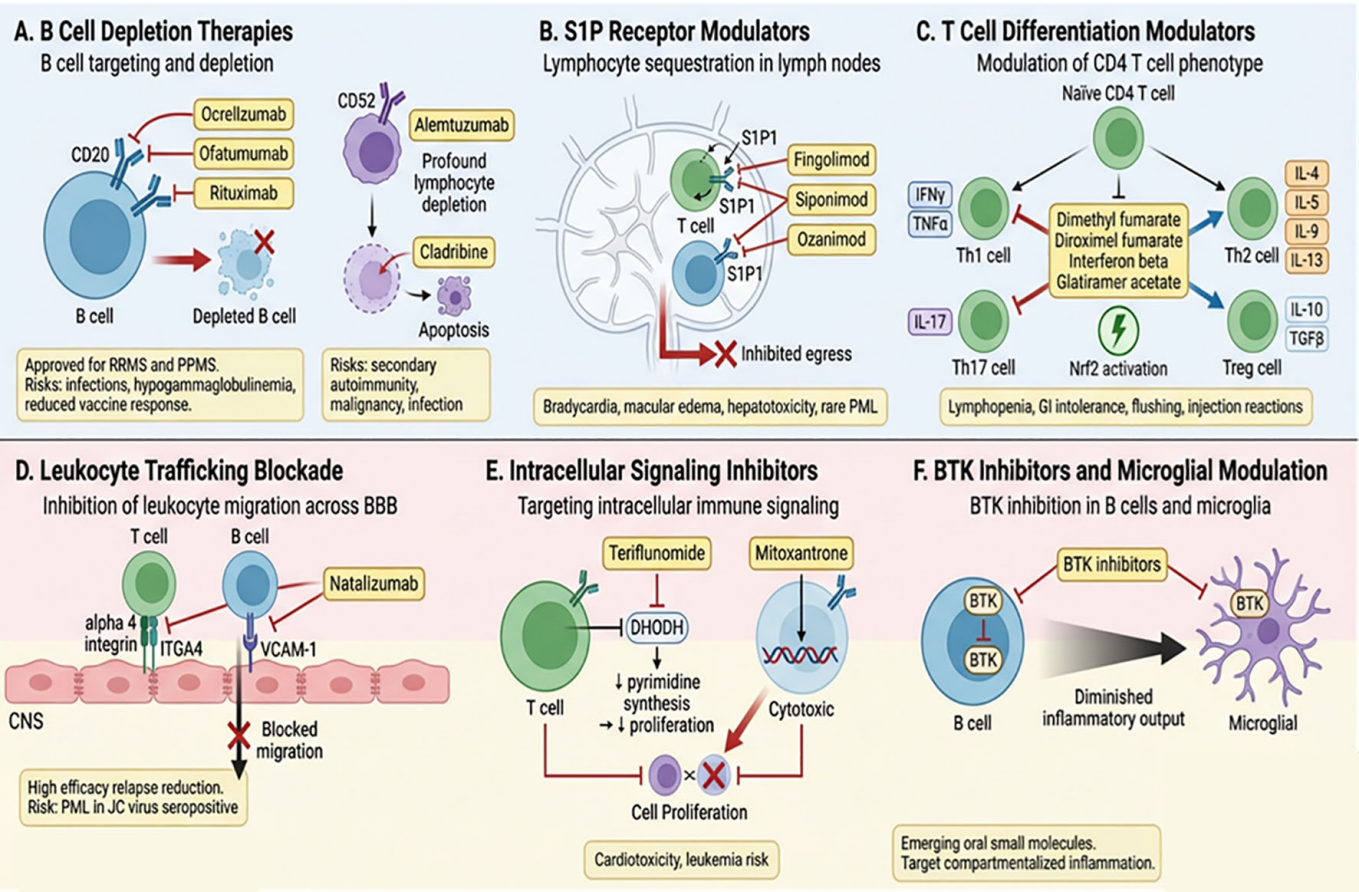
Mechanisms of current major disease-modifying therapy classes in multiple sclerosis management: **(A)** B-cell depletion therapies; **(B)** sphingosine-1-phosphate (S1P) receptor modulators; **(C)** T-cell differentiation modulators; **(D)** leukocyte trafficking blockade; **(E)** intracellular signaling inhibitors; and **(F)** Bruton's tyrosine kinase (BTK) inhibitors and microglial modulation.

Modern disease-modifying therapies (DMTs) for MS are broadly categorized based on their mechanisms of action, routes of administration, and efficacy profiles. These include immunomodulators, peripheral immune cell sequestrators, monoclonal antibodies, and immunosuppressants (**Figure 2**). Immunomodulators such as interferon-beta (IFN-β) and glatiramer acetate function by modulating immune cell behaviour without inducing broad immunosuppression. IFN-β, one of the earliest approved therapies (e.g., Avonex, Rebif, Betaseron), reduces inflammation by downregulating expression of MHC class II molecules, therefore, inhibiting T-cell activation, and decreasing BBB permeability (30, 31). Glatiramer acetate is a synthetic polymer that shares structural similarities with myelin basic protein and induces differentiation of anti-inflammatory Th2 and regulatory T (Treg) cells and inhibits Th1/Th17 responses (32, 33). Their clinical effect is limited despite their common use in relapsing-remitting MS (RRMS), with relapse rates decreasing by only 20–30% (34). Their tolerability is hampered by frequent injections and side effects such as flu-like symptoms (with IFN-β) and immediate post-injection reactions (with glatiramer). They do not prevent long-term disability progression.

A major advance has been the development of B-cell depleting therapies, which highlight the pathogenic role of B cells in MS. Ocrelizumab (Ocrevus) is a humanized anti-CD20 monoclonal antibody, authorized for RRMS and primary progressive MS (PPMS) (35). Other comparable medicines include subcutaneous ofatumumab (Kesimpta) and rituximab (36). Although these medicines are successful, they are associated with increased risk of infections, hypogammaglobulinemia and suboptimal vaccination responses and do not effectively target neurodegeneration. Immunosuppressants and cytotoxic agents (eg, alemtuzumab [Lemtrada], cladribine [Mavenclad]) have strong immunodepleting effects. Alemtuzumab is an anti-CD52 antibody and causes prolonged lymphocyte depletion followed by immunological reconstitution. Cladribine is a purine analogue that selectively depletes lymphocytes by apoptosis (37, 38). Risks of these agents include secondary autoimmunity, cancers, and infections. Their use is limited by rigorous monitoring requirements and patient selection criteria.

Oral sphingosine-1-phosphate (S1P) receptor modulators act by sequestering lymphocytes in lymph nodes and thereby reducing CNS infiltration. Fingolimod (Gilenya), the first oral DMT, binds to the S1P receptors (S1P_1_,_3_,_5_), resulting in internalization of the receptor and functional antagonism (39). Newer drugs, such as siponimod (Mayzent) and ozanimod (Zeposia), are more selective for the S1P_1_ and S1P_5_ receptors and have better safety profiles (40, 41). These medicines pose hazards such as bradycardia, macular oedema, hepatotoxicity and rare occurrences of progressive multifocal leukoencephalopathy (PML). Furthermore, they do not appreciably improve neuroprotection or remyelination. Monoclonal antibodies targeting immune cell trafficking, such as natalizumab (Tysabri), inhibit leukocyte migration across the BBB by blocking adherence of these cells to vascular endothelium through targeting alpha 4 integrin (ITGα4) (42). While natalizumab is highly effective (~70% relapse reduction), it carries a significant risk of PML, particularly in JC virus-seropositive individuals, and requires monthly intravenous administration with close monitoring.

Agents that modulate CD4 positive T cell differentiation and effector function constitute another major group of DMTs. Dimethyl fumarate and diroximel fumarate activate the nuclear factor erythroid 2 related factor 2 pathway and shift peripheral immunity away from pro inflammatory Th1 and Th17 responses toward a less inflammatory phenotype (43). This is accompanied by reduced production of cytokines such as IFN-γ and TNFα and relative enrichment of Th2 associated cytokines including IL-4, IL-5, IL-9, and IL-13 (44). IFN-β preparations and glatiramer acetate also act at the level of T cell differentiation, promoting regulatory and Th2 biased responses and decreasing trafficking of activated T cells into the central nervous system. These agents are generally considered safe and are widely used first line options, but their clinical efficacy is moderate and they are associated with adverse effects that include lymphopenia (45), gastrointestinal intolerance (46), flushing (44), flu like symptoms (47), and injection site reactions (48).

Therapies that directly limit leukocyte infiltration across the BBB act at another critical checkpoint in disease pathogenesis. Natalizumab is the prototypic agent in this category. This humanized monoclonal antibody binds alpha 4 integrin (ITGα4) on lymphocytes and monocytes, thereby blocking interaction with vascular cell adhesion molecule 1 (VCAM-1), on endothelial cells and preventing firm adhesion and transmigration into the central nervous system (42). Natalizumab is among the most effective approved DMTs, with substantial reductions in relapse rate and magnetic resonance imaging (MRI) activity, however its use is restricted by the risk of progressive multifocal leukoencephalopathy, particularly in individuals with antibodies to JC virus, and by the need for regular intravenous administration and intensive monitoring (49).

Agents that target intracellular signaling and neuroinflammatory pathways include teriflunomide, fumarates, Bruton tyrosine kinase inhibitors, and autologous hematopoietic stem cell transplantation. Teriflunomide inhibits dihydroorotate dehydrogenase, limiting *de novo* pyrimidine synthesis and thereby restricting proliferation of activated T and B lymphocytes (50). Older cytotoxic agents such as mitoxantrone, which intercalates DNA and inhibits topoisomerase II, provide broad suppression of proliferating immune cells but are now seldom used because of cumulative cardiotoxicity and risk of therapy related leukemia (51, 52). Experimental and early clinical data support the development of Bruton tyrosine kinase inhibitors as oral small molecules that modulate signaling in B cells and myeloid cells, including microglia, with the aim of attenuating chronic innate immune activation that drives compartmentalized inflammation and neurodegeneration within the central nervous system (53). In parallel, autologous hematopoietic stem cell transplantation seeks to reset the adaptive immune repertoire by ablating autoreactive lymphocyte clones and reconstituting a more tolerant immune system in carefully selected patients with aggressive disease (54, 55).

### 4.1 Key limitations of current DMTs

Despite their clinical utility, existing DMTs have several notable limitations. Most notably, they lack direct neuroprotective and remyelinating properties, focusing primarily on peripheral immune modulation. They do not sufficiently target the neurodegenerative aspect of MS, which is especially prominent in progressive forms of the disease (56, 57). Although some therapies such as ocrelizumab and siponimod have demonstrated modest benefits in progressive MS, the overall effect on disability progression is limited (18). Furthermore, the safety and tolerability profiles of DMTs are concerning, with risks including PML, secondary autoimmune disorder, cardiovascular effects and hepatotoxicity, requiring rigorous monitoring and often limiting patient adherence.

A further challenge is the highly variable individual response to DMTs, compounded by the lack of reliable predictive biomarkers. This variability translates into a trial-and-error approach to treatment selection (58). In addition, the high cost of DMTs limits access in many parts of the world, especially in low- and middle-income countries, worsening healthcare disparities. Routes of administration also influence adherence; injectable and infusion therapies are less convenient than oral options. Long term immune modulation can also impair host defence mechanisms, increasing infection susceptibility and complicating treatment sequencing, vaccination planning, and safety monitoring in people with MS.

### 4.2 The need for novel and complementary therapeutic strategies

Current DMT limitations highlight the urgent need for innovative therapeutics beyond immunosuppression to include neuroprotection, remyelination, mitochondrial support and immunological balance. Natural compounds such as flavonoids are being recognized for their multiple target, systems level mode of action. Differently from standard DMTs, flavonoids exert a simultaneous modulation of inflammation, oxidative stress, glial activation and regeneration signaling providing a more positive approach to MS treatment.

Flavonoids are a huge class of secondary metabolites that are found ubiquitously in fruits, vegetables, herbs and medicinal plants and are defined by their unique structure consisting of 15 carbon skeletons comprised of two benzene rings connected through a heterocyclic pyran ring (59, 60). This structural arrangement is responsible for not only their chemical stability, but also their vast spectrum of biological functions (61). Flavonoids are not only important for their antioxidant qualities, but also for their substantial influence on many physiological processes including regulation of gene expression, modulation of signal transduction and epigenetic alterations (62–64). These properties make flavonoids potential adjunct candidates for further MS research.

Flavonoids are of special interest in MS research because they can address multiple aspects of the disease at the same time (65). In contrast to classical pharmaceuticals that often target single routes or processes, flavonoids exert pleiotropic effects that are particularly well suited for the complex etiology of MS (5, 66). They influence immunological responses, reduce neuroinflammation, enhance neuroprotection and promote remyelination processes – all critical components in a comprehensive MS care (67). Moreover, their natural origin and typically positive safety profiles make them interesting alternatives or complements to conventional MS medications, which sometimes have severe side effects and limits (68).

Recent studies provide encouraging but uneven evidence that some flavonoid subclasses can modulate MS related inflammatory and repair pathways, mainly in experimental models. Special attention has been paid to glycosidic flavonoids, owing to their improved bioavailability and targeted delivery characteristics (69, 70). **Table 1** summarizes selected flavonoids that modulate MS relevant inflammatory, oxidative stress and repair related pathways in experimental or clinical studies. Compounds such as quercetin, rutin and hesperidin has ability to regulating molecular pathways in the development of MS like the NF-κB signaling pathway and the PI3K/Akt/mTOR cascade (86–89). These data suggest that flavonoids can influence disease relevant pathways in experimental models; whether this translates into altered MS progression remains unproven. A particularly intriguing area in this quest involves exploring natural flavonoids, which opens up possibilities for treatments that are both effective and conform to the growing patient demand for more natural, general healthcare approaches. Flavonoids also have beneficial pharmacokinetic and safety profiles (90), with many being orally accessible and well tolerated over long periods of time (91). Their ability to modulate the gut-brain axis is another layer of systemic immune modulation. Overall, these findings support biological plausibility in MS relevant models, but clinical efficacy and disease modifying activity remain unconfirmed unless supported by human MS studies (71).

**Table 1:** Summarizing findings of studies on various flavonoids and their potential effects in MS.

Flavonoid name	Flavonoid subclass	Type of study	Dose, route, duration, timing	Key findings	Evidence level^*^	Ref
Quercetin	Flavonol	Preclinical studies	50, 100 μg, I.P. route, every other day, for about 25 days	Reduced EAE severity and modulated gut brain axis related immune and metabolic pathways	MS specific preclinical evidence, mainly EAE and in vitro mechanistic evidence	(70)
Epigallocatechin-3-gallate (EGCG)	Flavanol	Clinical trials	Progressive MS: 1200 mg, Oral, daily for 36 months. Smaller RRMS study: 600 mg daily for 12 weeks	Nine clinical trials reviewed; Clinical trials of EGCG in MS show acceptable safety, but mixed or neutral effects on core MRI, relapse, disability and brain atrophy outcomes.	Human MS trial evidence	(71, 72)
Hesperidin	Flavanone	EAE mouse model	50 mg/kg/day, S.C. route, for 7 days, started 14 days after EAE induction with MOG and pertussis toxin	Reduced disease severity, limited leukocyte infiltration into the CNS, decreased pro-inflammatory cytokines, and increased regulatory T cells	MS specific preclinical evidence, EAE	(73)
Luteolin	Flavone	EAE mouse (SJL) model	35μM, 72 hours	Delayed onset and reduced severity of EAE, associated with decreased CNS inflammation and demyelination	MS specific preclinical evidence, EAE; additional in vitro immune cell evidence	(74)
Rutin	Flavonol	Cuprizone-induced demyelination mouse model	50 or 100 mg/kg, oral route, given during 0.2 percent cuprizone feeding for 6 consecutive weeks	Improved motor coordination, reduced demyelination, decreased oxidative stress markers, and lowered pro-inflammatory cytokine levels	Preclinical demyelination and repair evidence, cuprizone	(75)
Flavocoxid	Flavonoid	EAE mouse model	100 mg/kg, I.P. route, every other day, 14 days	Reduced clinical severity and modulation of Th1/Th17 differentiation	MS specific preclinical evidence, EAE	(76)
Baicalein	Flavone	Cuprizone-induced demyelination mouse model	100 mg/kg, intraperitoneal route, once daily, during the last 2 weeks of cuprizone exposure, from week 4 to week 6	Attenuated demyelination and suppressed neuroinflammation	Preclinical demyelination and repair evidence, cuprizone	(77)
Naringenin	Flavanone	Rotenone-induced neurotoxicity model	0.125%, 0.5%, and 2.0% w/w in diet, 4 weeks before MOG immunization; continued as an 8-week dietary intervention	Neuroprotective effects against neurotoxicity.	MS specific preclinical evidence, EAE	(78)
Kolaviron	Biflavonoid	Review of pharmacological properties	not available, review article.	Multiple health benefits, including anti-inflammatory effects	Review level or indirect pharmacological evidence only	(79)
Silymarin	Flavonolignan	Clinical trials	420 mg/day, oral, 6 months, given as adjunct treatment with interferon beta in RRMS patients	Antioxidant and anti-inflammatory properties, suggesting potential therapeutic benefits in MS	Adjunct human MS clinical evidence and immunomodulatory outcome, not established MS disease modifying evidence	(80)
Genistein	Isoflavone	EAE mouse model	Genistein 200 mg/kg, S.C. route, daily for 7 days, started 3 days after first clinical signs, around day 14 post immunization in established EAE	Reduced clinical severity, decreased pro-inflammatory cytokines, and promoted regulatory T cells	MS specific preclinical evidence, EAE	(81)
Daidzein	Isoflavone	EAE mouse model	0.22 g/kg, Oral, for 6 weeks.	Delayed onset and reduced severity of EAE, associated with decreased CNS inflammation.	MS specific preclinical evidence, EAE	(82)
Apigenin	Flavone	EAE mouse model	40 mg/kg, oral gavage, once daily, started at EAE onset and continued until day 15	Ameliorated clinical symptoms, reduced inflammatory cell infiltration, and decreased demyelination.	MS specific preclinical evidence, EAE	(83)
Kaempferol	Flavonol	Cuprizone demyelination model	40 µg/µL, intracerebroventricular, beginning of weeks 9 and 10 after 8 weeks of 0.2% w/w cuprizone feeding	Alleviated demyelination and microglial activation by inhibiting STAT3 and NFκB signaling	MS specific preclinical demyelination and repair evidence, cuprizone	(84)
Fisetin	Flavonol	Cuprizone demyelination model	80 mg/kg/day, oral, daily for 4 weeks starting in the second week of cuprizone induced	Reduced ferroptosis related injury and promoted remyelination in the cuprizone model	MS specific preclinical demyelination and repair evidence, cuprizone	(85)

^*^ ^Evidence level indicates whether the finding is based on human MS studies, EAE, cuprizone demyelination,^ *^in vitro^* ^systems, non MS neurological models, or indirect mechanistic evidence. Human MS studies are considered clinical evidence. EAE, cuprizone,^ *^in vitro^*^, and non MS findings are considered preclinical or indirect evidence and should not be interpreted as confirmed disease modifying or remyelinating efficacy in people with MS.^

### 4.3 Critical appraisal of strength and limitations of evidence

Flavonoids demonstrate consistent activity in experimental systems but the strength of evidence varies between compounds, models and outcomes. Most of the studies available use cell cultures, experimental autoimmune encephalomyelitis (EAE), cuprizone demyelination or other non-MS neuroinflammation models. These approaches are useful for testing immune, glial and oxidative stress mechanisms, but do not fully reproduce human MS. The induction of myelin reactive T cell immunity is the main effector of EAE. EAE is most useful for the study of inflammatory autoimmunity. Human MS is more complex, with B cells, CD8 T cells, compartmentalized CNS inflammation, cortical lesions, aging related repair failure and progressive neurodegeneration. The cuprizone model is valuable for the study of oligodendrocyte injury and remyelination, but as it is toxin driven and has limited peripheral adaptive immune involvement, positive results in this model primarily support effects on repair biology rather than proof of systemic MS immunotherapy.

Several experimental design issues also constrain translation. Many of the flavonoid studies use preventive dosing before or around MS induction, which is not representative of treatment of established MS. There is wide variation in doses, routes, treatment duration and formulations which makes comparison between studies difficult. Some studies are based primarily on clinical scores, cytokine levels or histology without pharmacokinetic data, measures of CNS exposure, long term disability outcomes or replication in independent laboratories. Reporting of randomization, blinding, power calculation and sex based analysis too is inconsistent. These issues bring in risk of bias and may lead to overestimation of efficacy, especially since negative or neutral preclinical findings are less likely to be published.

The clinical evidence is limited and mixed. Among the flavonoids discussed, EGCG has the most direct human data, but randomized studies have not demonstrated a clear benefit on core MS outcomes including MRI lesion activity, relapse rate, disability progression, or brain atrophy. Smaller studies may suggest effects on selected metabolic or symptom related measures but these findings should not be interpreted as evidence of disease modification. Evidence for quercetin, luteolin, apigenin, hesperidin, baicalein, naringenin and most other compounds is mainly preclinical or indirect. So clinical evidence should be distinguished from mechanistic plausibility. Thus, robust activity in cultured cells does not predict brain exposure or clinical efficacy. Future studies should employ standardized compounds, clinically relevant dosing, pharmacokinetic and pharmacodynamic endpoints, metabolite profiling, liver safety monitoring, sex and disease phenotype stratification, and clinically meaningful outcomes.

Accordingly, findings from EAE and cuprizone models are interpreted throughout this review as preclinical evidence supporting biological plausibility rather than as evidence of clinical efficacy in human MS. Clinical validation is considered present only when supported by studies conducted in people with MS. To make this distinction clearer, the revised manuscript separates evidence derived from *in vitro* systems, EAE studies, cuprizone demyelination models, other neurological or inflammatory models, and human MS trials. At present, EGCG has the most direct human MS evidence among the flavonoids discussed, although available clinical findings remain mixed. Most other compounds are supported mainly by preclinical, indirect or mechanistic evidence. To further compare translational relevance, selected flavonoids are ranked in **Table 2** according to MS specific evidence, relative preclinical efficacy, availability of human data, oral bioavailability, likely CNS exposure, safety considerations and therapeutic limitations. This ranking should not be interpreted as proof of clinical efficacy, because direct head to head clinical trials in people with MS are not available. Instead, it provides a practical framework for identifying candidates that may deserve priority in future pharmacokinetic and clinical studies.

**Table 2:** Comparative ranking of selected flavonoids according to translational promise in MS.

Rank^*^	Candidate	MS evidence and relative efficacy	BBB and CNS exposure	Therapeutic potential	Main limitation	Refs
1	EGCG	Most direct human MS evidence. Safe in trials, but no clear benefit on MRI lesions, relapse rate, disability or brain atrophy.	Limited and metabolite dependent CNS exposure.	Highest clinical readiness as an adjunct candidate.	Neutral clinical outcomes. Liver monitoring is needed at high supplemental doses.	(92–94)
2	Quercetin	Strong preclinical and mechanistic evidence in EAE and in vitro systems. Modulates inflammatory, oxidative stress and possible repair pathways.	Likely low to moderate and mainly metabolite dependent.	High mechanistic priority for future adjunct studies.	No robust human MS trial evidence. Circulating metabolites differ from free compound used in cell culture.	(70, 286)
3	Luteolin	Positive EAE and immune cell evidence. Targets inflammatory signaling and glial activation.	Possible CNS entry because of lipophilicity, but human CNS exposure is uncertain.	Moderate to high priority for immune and glial modulation.	Mainly preclinical evidence.	(74, 95)
4	Baicalein	Promising cuprizone and neuroinflammation data. Supports possible remyelination and barrier protective activity.	Preclinical CNS effects suggest possible exposure, but human MS exposure is unclear.	Moderate to high priority for repair focused studies.	No human MS trials. Strong dependence on model type.	(77, 96–98)
5	Hesperidin	EAE evidence supports reduced disease severity, lower CNS infiltration and cytokine modulation.	Uncertain and likely formulation dependent.	Moderate priority as a safe oral adjunct candidate.	Limited MS specific data and unclear CNS target engagement.	(73, 99)
6	Apigenin	Preclinical evidence supports reduced inflammatory infiltration and demyelination.	Possible but not well quantified in MS settings.	Moderate mechanistic value.	No clinical MS evidence and limited pharmacokinetic support.	(83, 100, 101)
7	Rutin	Cuprizone evidence suggests improved motor function, reduced demyelination and lower oxidative stress.	Likely limited passive CNS entry.	Moderate to low priority unless delivery is improved.	Evidence mainly from repair models and low CNS delivery confidence.	(75, 102)
8	Kaempferol	EAE evidence suggests reduced disease severity and cytokine modulation.	Uncertain and metabolite dependent.	Moderate early-stage candidate.	Mainly preclinical evidence and limited MS specific pharmacokinetic data.	(84, 103)

^*^ Ranking reflects translational promise, not proven clinical efficacy. The score considers MS specific evidence, consistency of preclinical findings, human data, oral bioavailability, likely CNS exposure, safety and feasibility for future clinical testing.

## 5 Systematic classification and structure-activity relationships of flavonoids

Flavonoids are a vast group of structurally diverse plant secondary metabolites, typically grouped into many major sub-classes based on their chemical structures and biosynthesis processes (104, 105). The most important classes include flavonols, flavones, flavanones, isoflavones, anthocyanidins, and flavanols with their characteristic structural characteristics and substitution patterns. Flavonols such as quercetin and kaempferol are based on a 3-hydroxyflavone skeleton with varying degrees of hydroxylation and methoxylation at positions 3, 5, 7, 3’ and 4’ (106–109). Flavones (e.g., apigenin and luteolin) do not have the 3-hydroxyl group but may have comparable substitutions, while flavanones (e.g., naringenin and hesperetin) have a saturated C-ring which influences their conformational flexibility and bioactivity (110–112).

Glycosylation significantly affects flavonoid variety and activity, resulting in glycosidic derivatives with increased water solubility and metabolic stability (113, 114). This modification is frequently at the 3-, 5- or 7-position of the flavonoid core, leading to the formation of O-glycosides by ether bonds and C-glycosides by carbon-carbon bonds. These glycosidic changes are known to be important for bioavailability, tissue distribution and receptor binding affinity (115). Sugar moieties can increase intestine uptake by active transport mechanisms, and also protect the aglycone against fast metabolism and excretion (116). Some glycosidic arrangements have also been identified in recent studies associated with improved capacity to pass the BBB, an important factor for neurological applications like MS therapy (117, 118).

The pharmacological importance of these structural aspects is particularly evident in view of their effects on MS-relevant pathways. Catechol B-ring-flavonoids have an increased potential to inhibit pro-inflammatory mediators such as cyclooxygenase-2 (COX-2) and inducible nitric oxide synthase (iNOS), while maintaining neuroprotective properties by activation of Nrf2 signaling (119). Some glycosidic flavonoids, especially those with C-glycosidic links, are very resistant to breakdown by β-glucosidase, allowing continuous release of active metabolites in target tissues (120, 121). This property is particularly advantageous in chronic disease such as MS when long-term treatment exposure is crucial for disease change.

## 6 Therapeutic goals and major molecular, cellular, and process-oriented targets in MS

A growing knowledge of MS as a dynamic interplay of autoimmune dysregulation, persistent neuroinflammation, demyelination and progressive neurodegeneration has changed the therapeutic goals. The current management strategies for MS have evolved beyond the classical immunosuppression to a multifaceted approach, addressing disease activity arrest, CNS repair promotion, and neurological function protection. The key therapeutic aims in MS are a number of interlinked targets. First and foremost, reduction of the autoimmune activation is essential to block the initial immunological responses that lead to the CNS inflammation mainly by the modulation of autoreactive CD4+ and CD8+ T cells, suppression of B cell activity and prevention of immune cell trafficking across the BBB (8, 9). Secondly, neuroprotection and prevention of neurodegeneration are focused on the later phases of the disease where oxidative stress, mitochondrial malfunction and excitotoxicity produce irreversible neuronal damage (57, 58). Third, OPCs must differentiate and mature to stimulate remyelination and myelin repair, which are essential for restoring neuronal conductivity and functional recovery (122).

Fourth, phenotypic conversion of glial cells, especially microglia and astrocytes from a pro-inflammatory (M1/A1) phenotype to an anti-inflammatory and neuroprotective (M2/A2) phenotype, is critical to resolve chronic inflammation and enhance tissue regeneration (20, 123). Fifthly, restoration of immunological homeostasis is becoming essential, through enhancement of regulatory T cell (Treg) function and generation of anti-inflammatory cytokines without global immunosuppression (14, 15). Sixth, BBB integrity enhancement is to stabilize tight junction proteins and suppress matrix metalloproteinase (MMP) activity to prevent the admission of peripheral immune cells (10, 124). Finally, targeting the gut-brain axis and microbiome has emerged as a novel therapeutic goal, with therapies targeted at restoring healthy microbial composition, boosting mucosal immunity, and indirectly modifying neuroinflammation (125, 126).

At the molecular level, therapeutic targets in MS include inflammatory signaling, oxidative stress responses, inflammasome activation, glial phenotype switching, oligodendrocyte survival and remyelination pathways. To avoid repetition, the flavonoid related modulation of NF κB, TLR/myeloid differentiation factor 88 (MyD88), JAK STAT, Nrf2, MAPK and inflammasome signaling (127–133) is discussed together in the integrated signaling section. Therapeutic treatments at the cellular level target modulation of important immunological and glial populations. CD4+ T helper cells (Th1/Th17) are important mediators of neuroinflammation, secreting IFN-γ and IL-17, respectively, and their inhibition is a major component of immunomodulatory therapy (10, 11). CD8+ cytotoxic T lymphocytes mediate axonal damage through direct cytotoxic pathways and are especially relevant in progressive MS (13). B cells and plasma cells are not only antibody-secreting cells but also participate in antigen presentation and cytokine generation, and their reduction has shown considerable therapeutic efficacy (17, 18). Microglia and macrophages are polarized into M1 (pro-inflammatory) and M2 (pro-repair) phenotypes, and the promotion of M2 states provides a mechanism of resolving inflammation (19, 20). Astrocytes are also capable of developing A1 neurotoxic or A2 neuroprotective characteristics and reprogramming them is emerging as a therapeutic target (21, 22). Lesions contain OPCs that typically fail to develop and are therefore prime targets for remyelination techniques (134). Regulatory T cells that are normally dysfunctional in MS may be possible agents for restoring immunological tolerance when properly enlarged or activated (14, 15).

In a broader sense, therapeutic objectives include biological processes beyond discrete molecules and cells, where oxidative stress and mitochondrial malfunction are significant contributions to dementia, and antioxidants and mitochondrial stabilizers are topics of study (58, 135). Besides epigenetic regulation (e.g. DNA methylation and histone modification) may also affect immune cell activation and gene expression in CNS, which provides other approaches of modulation such HDAC inhibitors and related drugs (136, 137). Autophagy and proteostasis are critical for brain health and the improvement of these processes has potential to treat protein aggregation and cell death (138, 139). Axonal transport and cytoskeletal integrity are compromised in MS and neuroprotective compounds that stabilize microtubules and inhibit calpain-mediated degradation are under development (140, 141). Finally, gut-brain-axis signaling by way of microbial metabolites such as short-chain fatty acids can influence immune responses in the peripheral system and the CNS (125, 126).

## 7 Flavonoids’ anti-inflammatory properties in multiple sclerosis pathology

The anti-inflammatory properties of flavonoids are remarkably precise in targeting the complex inflammatory cascades that are central to MS pathology. At the molecular level, flavonoids exert their effects through multiple interconnected mechanisms, starting with modulation of NF-κB signaling, one of the master regulators of inflammation in MS. Specific flavonoid subclasses, especially flavones and flavonols, directly inhibit IκB kinase (IKK) activity, thereby preventing phosphorylation and subsequent degradation of IκBα (142). This intervention effectively sequesters NF-κB dimers in the cytoplasm, blocking their translocation to the nucleus and the subsequent transcription of pro-inflammatory genes including TNF-α, IL-1β, and IFN-γ. Importantly, glycosylated derivatives of apigenin and luteolin show enhanced potency in this regard, achieving significant suppression of inflammatory cytokine production at nanomolar concentrations (143).

In addition to the modulation of NF-κB, flavonoids also target multiple other pathways crucial for the regulation of neuroinflammation in MS. The JAK/STAT pathway, which is hyperactivated in MS patients, is another important target. Chrysin and its glycosidic derivatives specifically inhibit JAK2 phosphorylation, preventing the activation of STAT3 and the subsequent upregulation of genes involved in T-cell activation and differentiation (144). This mechanism is particularly pertinent to the suppression of the development of Th17 cells, a key driver of MS pathogenesis. Concurrently, some flavonoids activate suppressor of cytokine signaling (SOCS) proteins, thereby providing an additional layer of negative feedback regulation on JAK/STAT signaling (145).

Oxidative stress constitutes another critical facet of MS pathology wherein flavonoids exhibit potent protective effects. Beyond direct antioxidant activity, flavonoids modulate redox-sensitive signaling cascades implicated in neuroinflammation (146). Nrf2 activation by certain flavonoid subclasses results in enhanced expression of antioxidant response element (ARE)-regulated genes including heme oxygenase-1 (HO-1) and NAD(P)H quinone dehydrogenase 1 (NQO1) (147). The spatial and temporal regulation of these anti-inflammatory effects indicates sophisticated orchestration of distinct flavonoid mechanisms. For example, the early phase of flavonoid action features rapid suppression of acute inflammatory mediators via direct enzyme inhibition and receptor antagonism. Later phases involve longer-term epigenetic changes including histone deacetylase (HDAC) inhibition and DNA methyltransferase (DNMT) modulation which contribute to reprogramming inflammatory gene expression patterns (148, 149). Clinical relevance of these mechanisms becomes evident when considering the differential effects of various flavonoid subclasses on specific MS subtypes. RRMS patients responds particularly well to flavonoids targeting NF-κB and MAPK pathways due to their prominent role in relapse-associated inflammation (150). By contrast, progressive forms of MS benefit more from flavonoids that modulate mitochondrial function and promote neuroprotection via Nrf2 activation and mTOR inhibition (6).

The ability of flavonoids to target these multiple anti-inflammatory pathways is what makes them so remarkably versatile in targeting the complex immunopathology of MS. Flavonoids do not target single components of the inflammatory cascade, but instead, they interact with multiple nodes in the network to achieve effects that enhance their overall therapeutic impact. This multi pathway activity provides a useful mechanistic rationale for further study, but whether it leads to sustained clinical benefit in MS remains unproven and requires stronger human evidence. These anti-inflammatory mechanisms are supported mainly by preclinical and mechanistic evidence. They should be interpreted as pathway level support for biological plausibility rather than proof of clinical efficacy in people with MS.

## 8 Key pathways implicated in multiple sclerosis pathogenesis

**Figure 3:**
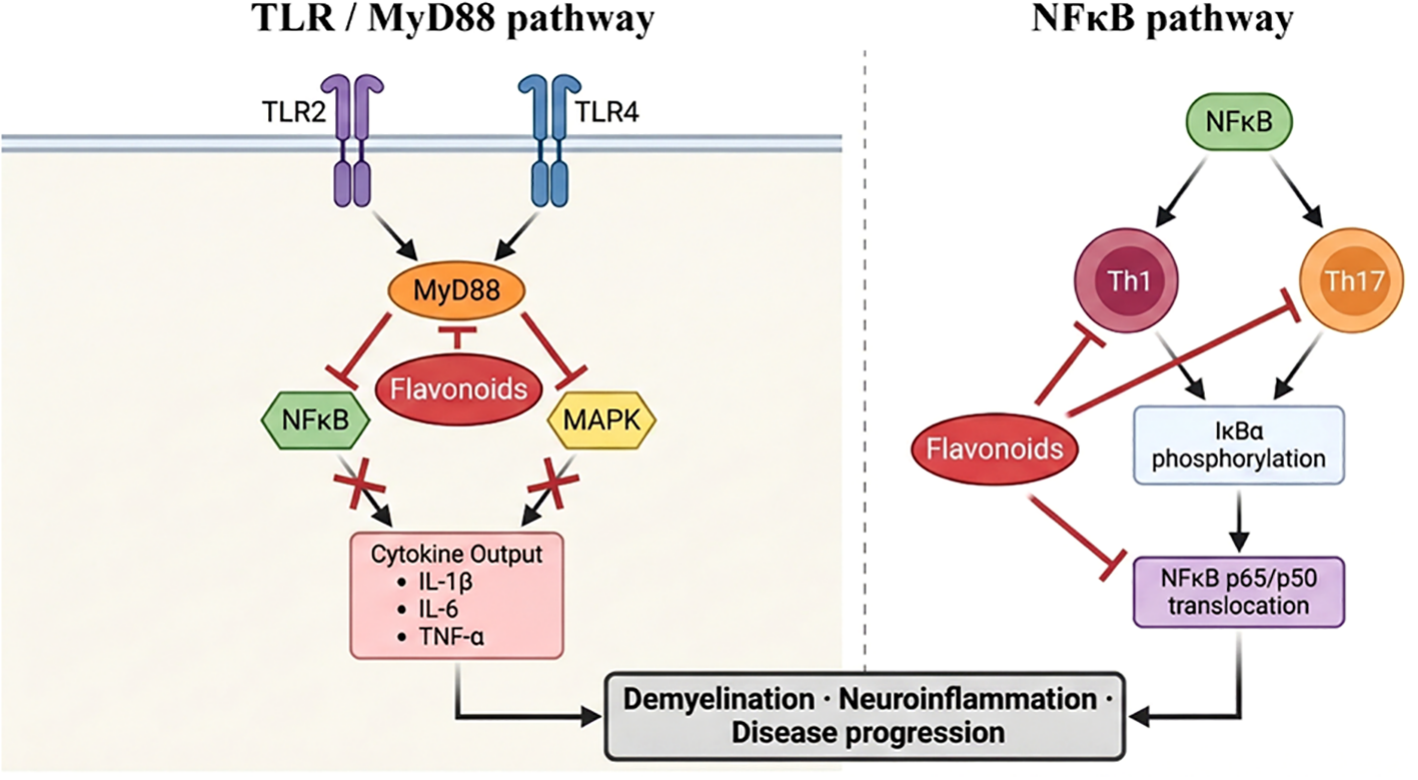
Integrated inflammatory and redox signaling pathways targeted by flavonoids in MS relevant models. TLR2 and TLR4 activation recruits MyD88 and downstream IRAK TRAF6 TAK1 signaling, leading to NF κB and MAPK activation and increased production of IL 1β, IL 6, TNF α and other inflammatory mediators.

Two key pathways implicated in MS pathogenesis the TLR/MyD88 pathway and the NF-κB pathway (**Figure 3**) have emerged as potential targets for flavonoid-mediated intervention.

### 8.1 Flavonoids and TLR/MyD88 pathway

The TLR/MyD88 signaling pathway plays a pivotal role in initiating and sustaining inflammatory responses in MS. Some TLRs, in particular TLR2 and TLR4, are over-expressed in MS patients and in EAE models, playing a role in the progression of the disease by promoting the production of pro-inflammatory cytokines and the differentiation of Th17 cells (151). MyD88, a critical adaptor protein downstream of most TLRs, activates the NF-κB and mitogen-activated protein kinase (MAPK) pathways which culminate in the production of inflammatory mediators, such as IL-1β, IL-6 and TNF-α (152). Flavonoids such as quercetin and EGCG have been demonstrated to control this quite well. Quercetin suppresses TLR4 signaling by preventing the binding of MyD88 with IRAK1, leading to decreased activation of NF-κB and MAPK pathways (153). In the same vein, EGCG, one of the major catechins present in green tea, decreases the expression of TLR4 and MyD88-dependent signaling and thereby reduces the production of pro-inflammatory cytokines in EAE models (154). These data demonstrate that flavonoids could suppress neuroinflammation through the TLR/MyD88 pathway, representing a potential strategy to the treatment of MS. In addition to quercetin and EGCG, several flavonoids modulate the TLR4 MyD88 signaling pathway, notably genistein reduces EAE severity while altering the spinal cord Toll like receptor, and it directly suppresses microglial TLR4 MyD88 signaling by limiting LPS TLR4 engagement and downstream NF-κB activation (155, 156). Also, baicalin improves symptoms and tissue damage in EAE and directly blocks the CD14-dependent TLR4–MyD88–NF-κB signaling pathway in models of CNS inflammation (96, 97). Similarly, Nobiletin attenuates EAE through limiting Th17 differentiation and related cytokines, and a nobiletin derivative has been shown to downregulate TLR4 MyD88 MAPK signaling and STAT3 activation in microglia, supporting mechanism-based convergence on this axis (157, 158). These findings are mainly derived from EAE, CNS inflammation, or mechanistic models.

### 8.2 Flavonoids and NF-κB pathway

NF-κB is a major transcription factor that controls inflammatory genes and is overactive in MS patients, causing higher levels of pro-inflammatory cytokines and chemokines (159, 160). Activation of NF-κB in microglia, astrocytes and infiltrating immune cells exacerbates neuroinflammation and promotes demyelination (161). NF-κB signaling is also required for development and activity of Th1 and Th17 cells that are significant players in MS pathophysiology (162, 163). Flavonoids are potent inhibitors of the NF-κB pathway. Curcumin is a polyphenolic curcuminoid from turmeric, which suppresses NF-κB activation by inhibiting phosphorylation and degradation of IκBα, and consequently nuclear translocation of p65 and p50 subunits (164). Curcumin administration dramatically reduced clinical ratings and infiltration of inflammatory cells into the central nervous system in EAE models, indicating its therapeutic potential (165). Another flavonoid, apigenin, was proven to inhibit NF-κB signaling and downregulate IL17A and IL23, thus inhibiting Th17-mediated inflammation (100). These findings show that these flavonoids are able to alter NF-κB signaling, which can be a possible strategy to alleviate symptoms of MS. These observations support mechanistic relevance of NF κB modulation, but clinical benefit in people with MS remains unproven.

## 9 Neuroprotective effects of flavonoids in multiple sclerosis: cellular and molecular mechanisms

**Figure 4:**
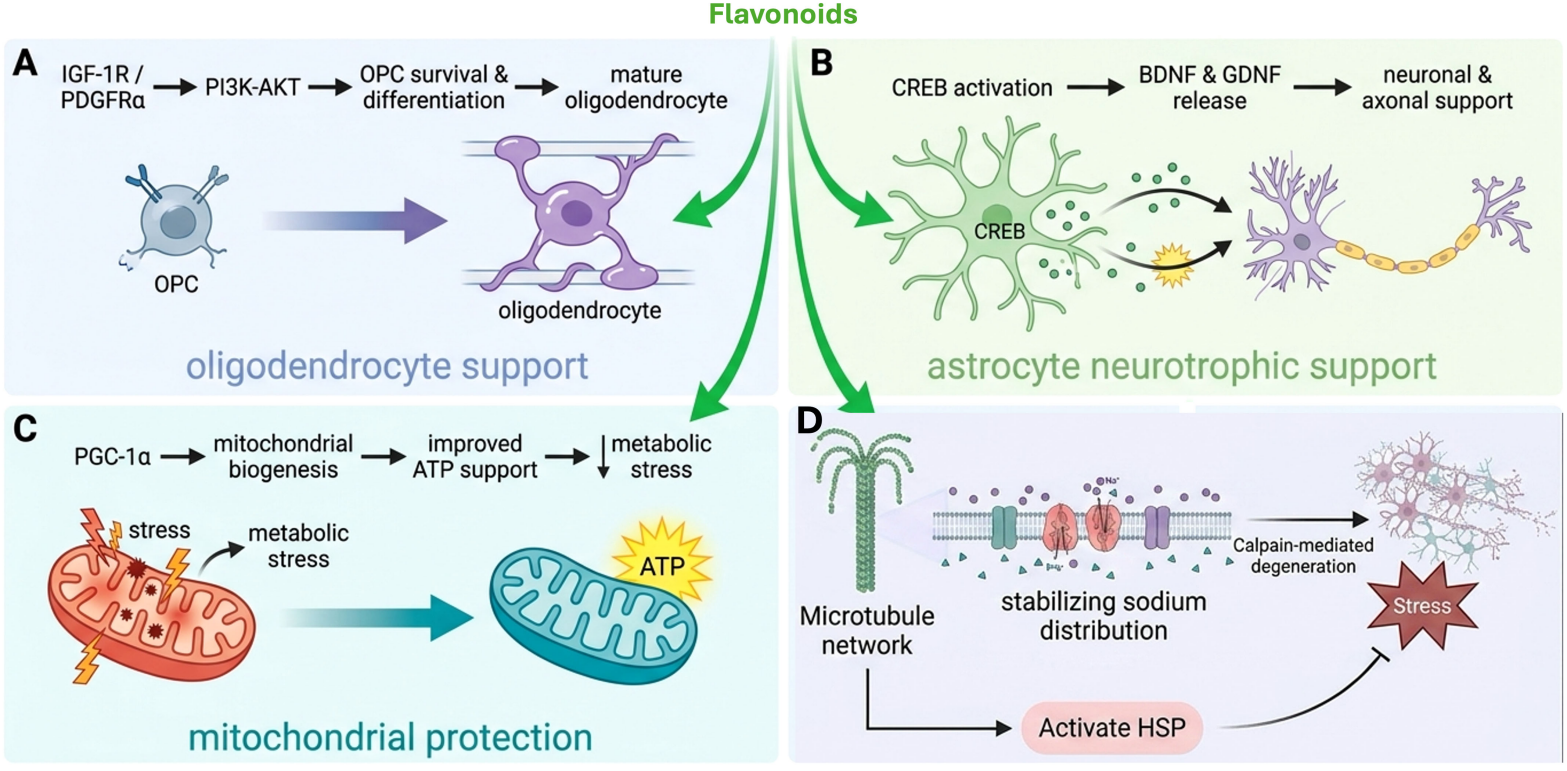
Cellular and molecular mechanisms of flavonoid-induced neuroprotection in MS (Preclinical evidence). Flavonoids target several CNS pathways that are involved in MS pathology: **(A)** PI3K–Akt activation through IGF-1R and PDGFRα promotes neuroprotection by inducing differentiation of oligodendrocyte precursor cells (OPCs) and reducing microglial activation. **(B)** Flavonoids modulate astrocytes by increasing the levels of neurotrophic factors (BDNF, GDNF) and CREB phosphorylation, which enhances neurotrophic support and reduces astrogliosis. **(C)** Flavonoids protect mitochondria by stimulating PGC1α-dependent mitochondrial biogenesis, which improves ATP production, oxidative metabolism and resistance to metabolic stress. **(D)** Flavonoids protect axons by stabilizing microtubules, maintaining the distribution of sodium channels and attenuating calpain-mediated axonal degeneration, in part via the activation of heat shock proteins (HSP).

Flavonoids mediate neuroprotection in MS through complex interactions with neuronal cells, glial cells and the wider neurovascular unit. At a cellular level, certain subclasses of flavonoids display a high degree of specificity in the activation of survival pathways while also suppressing neurodegenerative processes (**Figure 4**). For example, glycosylated derivatives of baicalein and wogonin activate PI3K/Akt signaling in OPCs specifically, promoting their differentiation into mature myelinating oligodendrocytes. This is accomplished by directly binding to insulin-like growth factor-1 receptor (IGF-1R) and platelet-derived growth factor receptor alpha (PDGFRα), which then activates downstream signaling cascades that promote OPC survival and proliferation (166, 167).

Another significant element of flavonoid-mediated neuroprotection is regulation of astrocytes. Orientation and its glycosidic derivatives promote astrocytic production of brain derived neurotrophic factor (BDNF) and glial cell line derived neurotrophic factor (GDNF) through cyclic AMP response element-binding protein (CREB) phosphorylation (168). These neurotrophic factors increase neuronal survival and stimulate axonal regeneration and prevent the neurodegenerative processes typical of progressive MS. Critically, the efficiency by which these flavonoids penetrate the BBB is dictated by their glycosylation pattern, with C-glycosides demonstrating better BBB penetration compared to O-glycosides (116).

Flavonoid intervention is particularly aimed at mitochondrial dysfunction, a hallmark of MS pathology. Scutellarin and its derivatives have unique capabilities in mitochondrial membrane stabilization, permeability transition pore opening blocking and ATP production preservation. These effects are achieved through different mechanisms including direct interaction with mitochondrial permeability transition pore components, up-regulation of factors involved in mitochondrial biogenesis such as peroxisome proliferator-activated receptor gamma coactivator 1-alpha (PGC-1α) and increasing the efficiency of oxidative phosphorylation (169). To keep the neural energy supply intact and to avoid excitotoxicity-induced cell death, mitochondrial function must be maintained.

The most clinically significant part of flavonoid neuroprotection in MS may be axonal protection. In example, vitexin derivatives have been found to be effective in maintaining axonal integrity through several routes (170). These methods include direct binding to tubulin to stabilize microtubule networks, inhibition of calpain-mediated spectrin degradation and maintenance of Na channel distribution along axonal membranes. Additional flavonoids promote the expression of heat shock proteins (HSPs) in neurons and so improve defense against proteotoxic stress and promote correct protein folding during inflammatory episodes (171). Flavonoid intervention provides full support to the neurovascular unit comprising endothelial cells, pericytes, astrocytes and neurons. Flavonoids improve the integrity of the BBB by numerous mechanisms, such as increased expression of tight junction proteins (claudin-5, occludin) and regulation of matrix metalloproteinase (MMP) activity (172). This vascular protection is especially critical to avoid immune cell invasion and preserve CNS homeostasis. Furthermore, flavonoids increase angiogenesis and vascular remodeling through the regulation of the vascular endothelial growth factor (VEGF) pathway, ensuring sufficient nutrition and oxygen delivery to the afflicted brain areas (173).

Emerging evidence suggests that epigenetic control is a critical mechanism driving flavonoid mediated neuroprotection relevant to MS, where persistent neuroinflammation and oxidative stress may promote maladaptive transcriptional patterns. Specific flavonoid subclasses such as methoxylated flavones including wogonin may influence class I histone deacetylase pathways that are core components of HDAC repression complexes, supporting chromatin level remodeling and altered gene expression patterns (174). Such transcriptional reprogramming agrees with downstream effects on antioxidant and synaptic plasticity related pathways in the central nervous system (175). Flavonoids also modulate non coding RNA networks including microRNAs associated with neuronal survival and regeneration. For example, quercetin can regulate microglia derived exosomal microRNA cargo such as let 7e 5p, with downstream effects on neural stem cells and hippocampal neurogenesis (176).

Synaptic plasticity and cognitive preservation are particularly relevant in MS, given the substantial burden of cognitive impairment (177). Consistent with a pro plasticity profile, brain available quercetin conjugates such as quercetin 3 O glucuronide have been shown to rescue basal synaptic transmission deficits and long-term potentiation in hippocampal slices from a transgenic mouse model (178). These neuroprotective mechanisms are supported mainly by preclinical, *in vitro*, and indirect neurological evidence. They provide mechanistic rationale for further study but do not establish neuroprotective efficacy in people with MS.

## 10 Flavonoids can promote microglial M2 activation

**Figure 5:**
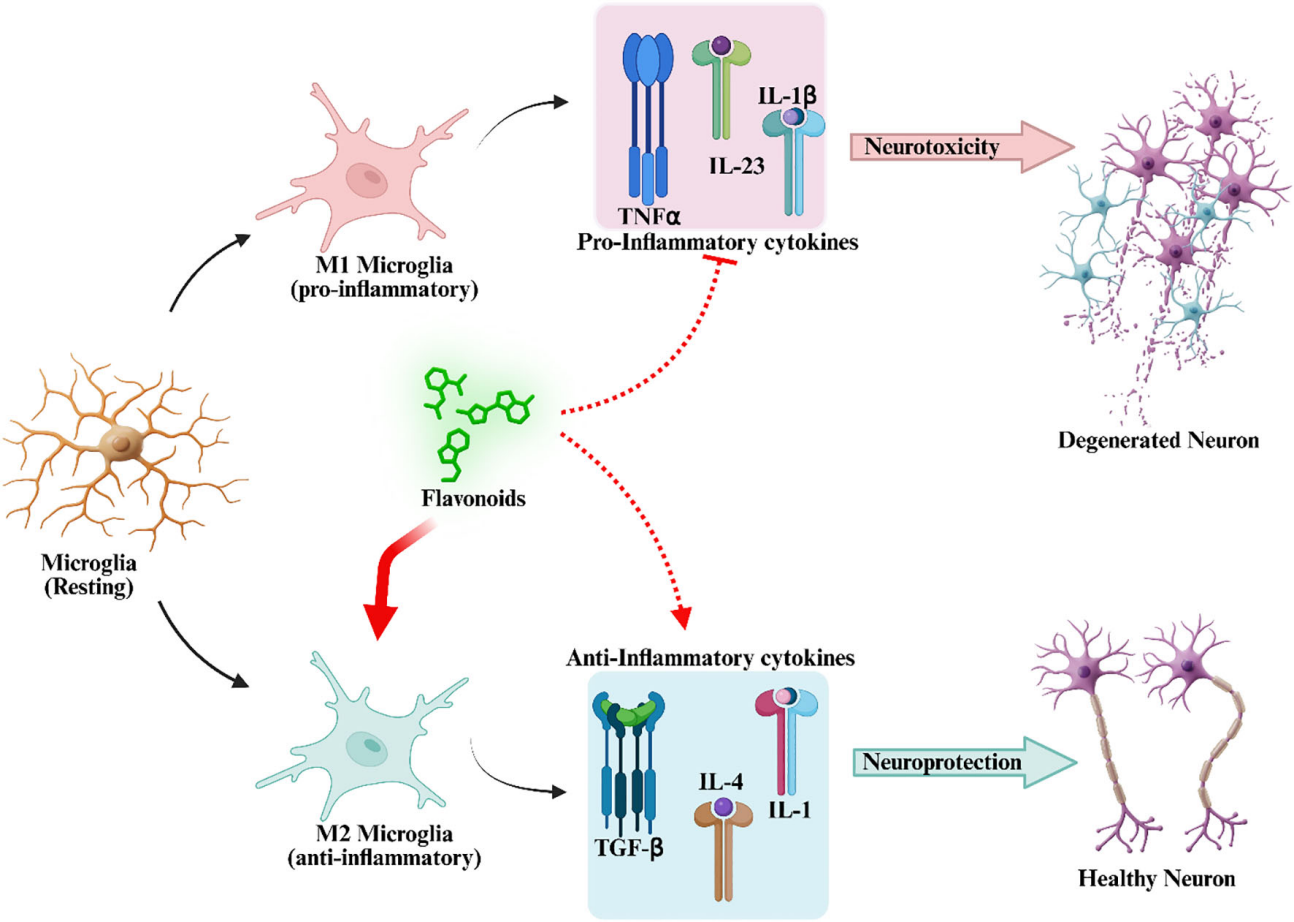
Flavonoid modulation of microglial phenotype and repair permissiveness in MS preclinical models (Preclinical evidence). Pro inflammatory M1 like microglia produce iNOS, TNF α, IL 1β, IL 6 and reactive oxygen species, which support demyelination, axonal injury and inflammasome activation.

Microglial cells, the resident immune cells of the central nervous system, respond to flavonoid treatment through complex polarization dynamics such as shifting M1 (proinflammatory) to M2 (anti-inflammatory) which results in neuroprotective effect (**Figure 5**). Flavonoids such as isovitexin and the flavanone naringenin shift activated microglia from a classical M1 profile toward an alternative M2 state, with reduced inducible nitric oxide synthase and pro inflammatory cytokines together with increased arginase 1 and IL-10, through activation of CaMKKβ AMPK PGC 1α and modulation of MAPK signaling respectively (179). Naringenin also suppresses the NLRP3 inflammasome in microglia, limiting IL-1β and IL-18 release in *in vitro* and *in vivo* models (180). Baicalin further restrains microglia driven inflammation by suppressing NLRP3 together with TLR4 NF-κB signaling, while quercetin promotes mitophagy to lower mitochondrial reactive oxygen species and thereby attenuate NLRP3 activation in microglia (181, 182). The resulting M2 biased microglial phenotype supports debris clearance and fosters oligodendrocyte progenitor differentiation and remyelination, processes that are central to lesion repair in demyelinating disease (183).

The flavonoid glycoside rutin has been shown to alter microglial and macrophage activation toward an anti-inflammatory CD150/CD206 M2 phenotype, suggesting a potential involvement in neuroinflammatory control (102). Similarly, a greater diversity of plant-derived flavonoids can alter macrophage polarization from the pro-inflammatory M1 phenotype to the anti-inflammatory M2 phenotype, thereby helping to maintain immunological homeostasis (184). Flavonoids regulate oxidative stress and gut microbiota, which is of therapeutic potential through mechanisms such as superoxide dismutase activation and epithelial barrier repair (185). Inhibition of histone deacetylase (HDAC) and activation of p38 MAPK by these further confirm their roles in immune modulation and illness management. **Table 3** summarizes flavonoids reported to promote M2 like microglial activation in preclinical or mechanistic systems. These findings support repair related biological plausibility but do not establish clinical efficacy in MS.

**Table 3:** List of flavonoids that promote microglial M2 activation.

Flavonoid	Dietary sources	Mechanism of M2 activation	Reference
Quercetin	Apples, onions, berries, tea	Modulates NF-κB and STAT6 pathways; increases IL-10 and TGF-β production.	(186)
Luteolin	Celery, parsley, green peppers, chamomile tea	Inhibits pro-inflammatory cytokines (TNF-α, IL-6); upregulates arginase-1 and IL-4.	(95)
Apigenin	Chamomile, celery, parsley	Activates PPARγ pathway; suppresses M1 markers; enhances CD206 and YM1 expression.	(101)
Baicalein	Scutellaria baicalensis (Chinese skullcap)	Reduces oxidative stress; promotes anti-inflammatory genes via AMPK/Nrf2 pathway.	(98)
EGCG	Green tea	Modulates TLR4/NF-κB pathway; enhances IL-10 production.	(187)
Naringenin	Citrus fruits (grapefruit, oranges)	Activates PI3K/Akt/mTOR pathway; increases Arg1 and Fizz1 expression.	(180)
Kaempferol	Kale, beans, spinach, broccoli	Inhibits JAK/STAT1 pathway; enhances STAT6 pathway; promotes M2 polarization.	(103)
Hesperidin	Citrus fruits (oranges, lemons)	Modulates MAPK and NF-κB pathways; reduces neuroinflammation.	(99)
Rutin	Buckwheat, apples, citrus fruits	Scavenges ROS; upregulates anti-inflammatory mediators.	(102)
Myricetin	Berries, grapes, walnuts	Inhibits pro-inflammatory cytokines; enhances M2-associated gene expression.	(188)
Cyanidin	Red berries (cherries, raspberries, blackberries)	Activates AMPK pathway; reduces inflammatory responses in microglia.	(189)
Genistein	Soybeans, legumes	Modulates estrogen receptor signaling; suppresses pro-inflammatory pathways.	(190)

## 11 The role of flavonoids in multiple sclerosis recovery

**Figure 6:**
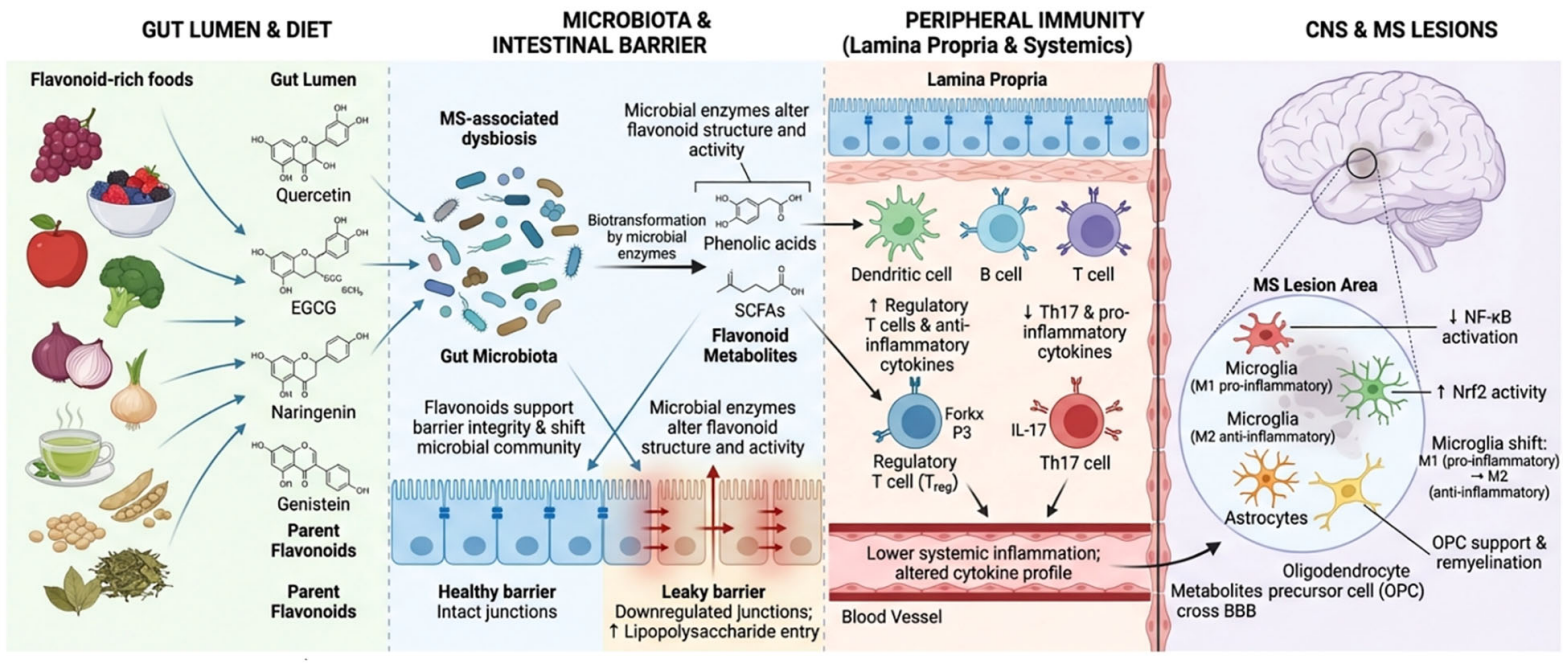
A conceptual pathway that shows how dietary flavonoids are transformed by the microbiota into immune modulation, and reaches the CNS to influence MS pathology.

**Figure 7:**
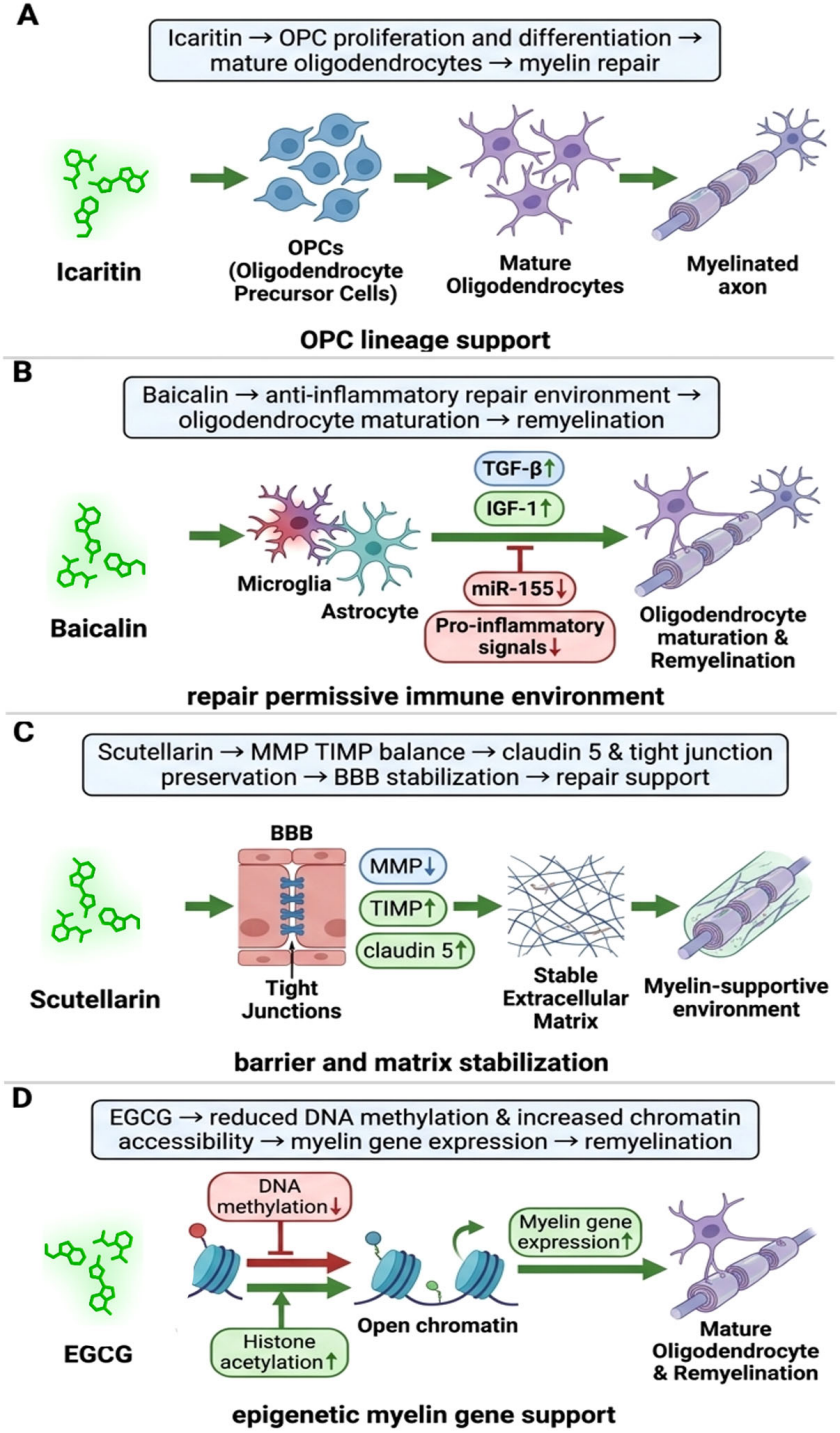
Schematic overview illustrating the neuroprotective and pro-remyelinating actions of selected flavonoids in MS models (Preclinical evidence). **(A)** Icariin enhances OPC recruitment, proliferation, and differentiation, supporting oligodendrocyte lineage expansion. **(B)** Baicalin exerts anti-inflammatory and antioxidant effects by shifting the cytokine balance toward anti-inflammatory mediators (↑TGF-β, ↑IGF-1) while suppressing pro-inflammatory signaling (↓miR-155), and promotes remyelination and motor–cognitive recovery. Baicalin upregulates pro-myelinating microRNAs miR-219 and miR-338 that drive OPCs differentiation and myelin gene expression. **(C)** Scutellarin promotes a pro-myelination environment in the extracellular matrix by restoring the MMP–TIMP balance (↓MMPs, ↑TIMPs) and reinforcing BBB integrity through increased tight junction proteins (e.g., claudin-5), protecting BBB structure and limiting neuroinflammation. Together, these flavonoids work through complementary cellular, molecular, epigenetic, and barrier-protective mechanisms to support myelin repair in MS. **(D)** EGCG modulates epigenetic regulation of myelination by inhibiting DNA methyltransferases and histone deacetylation, leading to chromatin relaxation, reactivation, and epigenetic upregulation of myelin-associated genes, thereby facilitating remyelination in cuprizone-induced demyelination models.

In MS, remyelination of damaged myelin sheaths has emerged as a critical therapeutic goal that can result in functional recovery and disease stabilization. Flavonoids have emerged as potent agents in promoting remyelination across cellular and molecular levels, beginning with direct stimulation of OPCs to enhance proliferation, migration, and differentiation into mature myelinating oligodendrocytes (**Figure 6**). Icaritin simultaneously increases OPC proliferation and differentiation and augments myelin formation in the cuprizone model (191) (**Figure 7A**). Baicalin directly promotes central nervous system remyelination via PPARγ signaling and promotes oligodendrocyte maturation in complementary *in vivo* and *in vitro* systems (192). Baicalein improves motor and cognitive outcomes and promotes remyelination in a MS model via antioxidant mechanisms that maintain mitochondrial function and promote oligodendrocyte lineage development (193) (**Figure 7B**). Scutellarin alleviates cuprizone induced demyelination by improving mitochondrial dysfunction in microglia and reducing inflammatory signaling, thereby creating a favourable environment for myelin repair (194) (**Figure 7C**).

At the molecular level, flavonoids have interactions with myelin-associated signaling relevant to remyelination. Catechin flavanols such as EGCG inhibit DNA methyltransferases and several flavonoids affect histone deacetylases, changes consistent with epigenetic upregulation of myelin genes, and *in vivo* icariin and baicalin increase myelin basic protein and enhance remyelination in cuprizone demyelination models (195–197) (**Figure 7D**). Astrocyte oligodendrocyte crosstalk is reinforced by flavonoids, with astrocytic BDNF signaling shown to support differentiation and maturation, and flavonoid studies aligning with CREB-dependent neurotrophic support from astroglia (193, 198, 199). The extracellular matrix milieu is also tuned by flavonoids; scutellarin-rich preparations reduce MMP activity and preserve barrier tight junctions, consistent with a balanced MMP to TIMP state that favors myelin repair, and claudin-5 focused work underscores the central role of tight junction restoration for barrier integrity during repair (200, 201).

Microglial contributions are shaped by flavonoids, with baicalein shifting cytokine profiles toward anti-inflammatory programs that include increased TGFβ, and complementary studies establishing IGF-1 signaling in microglia as a driver of pro repair phenotypes during resolution of neuroinflammation (202–205). Axonal preservation during remyelination is supported by vitexin, which regulates heat shock protein expression under stress, while spectrin-based axonal scaffolds and their vulnerability to calpain proteolysis emphasize how stabilizing axonal architecture preserves sodium channel clustering and excitability needed for effective remyelination (206–208). Delivery technologies now improve central exposure, with baicalin liposomes engineered for brain targeting and quercetin or EGCG nano formulations showing enhanced transport across barrier models while maintaining bioactivity (209–211). At the genetic layer, micro-RNA programs critical for myelin repair are engaged, miR-219 and miR-338 promote oligodendrocyte differentiation, and flavonoids such as baicalin suppress pro inflammatory miR-155 in microglia, creating a permissive environment for remyelination (212–214). Finally, flavonoids increase energy metabolism during the high demand phase of myelin synthesis by engaging PGC-1α mediated mitochondrial biogenesis and ATP production in neurons, a process repeatedly documented for quercetin and central to sustaining myelin formation (67, 215). These remyelination related findings are mainly derived from cuprizone, EAE, *in vitro*, and other preclinical models. They support remyelination permissiveness and repair biology, but confirmed remyelination efficacy in people with MS has not yet been established.

## 12 Combination therapy: evidence, cautions and hypotheses

Combining flavonoids with approved DMTs is a biologically plausible adjunct strategy in MS, but current evidence does not yet establish consistent clinical synergy. The rationale is based on complementary mechanisms, since many approved therapies mainly reduce peripheral immune activation or immune cell trafficking, whereas selected flavonoids may influence oxidative stress, NF κB signaling, Nrf2 activity, mitochondrial injury, glial activation and repair related pathways. Therefore, flavonoid and disease modifying therapy combinations should be interpreted as investigational approaches unless supported by controlled clinical trials with clear MRI, relapse, disability and safety outcomes.

Some clinical studies suggest possible adjunctive effects, but these findings remain preliminary. In relapsing remitting MS patients receiving interferon beta, quercetin supplementation at 500 mg daily for 6 months was reported to improve antioxidant enzyme activity and reduce inflammatory cytokines, including TNF alpha and IL 6, with modest changes in EDSS scores (67). These results suggest possible biomarker level benefit, but they do not prove clinical synergy or sustained disease control. Similarly, coadministration of silymarin at 420 mg daily with fingolimod at 0.5 mg daily was associated with reduced increases in ALT and AST without worsening neurological outcomes (66). This finding is better interpreted as a possible hepatoprotective or tolerability related effect, rather than evidence that silymarin improves the disease modifying efficacy of fingolimod.

Mechanistic studies provide additional support for combination hypotheses. Fingolimod and quercetin have been reported to jointly suppress NF κB signaling, reduce reactive oxygen species and support oligodendrocyte survival and remyelination related gene expression in microglial and oligodendrocyte models (216). Pharmacokinetic studies also suggest that flavonoid glycosides can affect cytochrome P450 enzymes and transporters such as P glycoprotein and BCRP, which may alter drug exposure and tissue distribution (217). However, these effects should be viewed with caution. Transporter and enzyme modulation may improve exposure in some settings, but it may also create unpredictable food drug or supplement drug interactions with approved MS therapies. Therefore, such interactions require formal pharmacokinetic and safety testing before they can be considered clinically useful.

A second mechanistic example involves dimethyl fumarate and flavonoid mediated activation of the Nrf2 antioxidant pathway. Dimethyl fumarate activates Nrf2 mainly through modification of Keap1 cysteine residues, leading to Nrf2 nuclear translocation and transcription of antioxidant response genes (218). In contrast, some flavonoids may stabilize Nrf2 through Keap1 independent mechanisms, including GSK 3β inhibition and enhanced recruitment of CREB binding protein to antioxidant response element promoters (219). Combination studies using dimethyl fumarate and luteolin have reported increased p GSK 3β, Nrf2 nuclear translocation and antioxidant gene expression, suggesting a possible dual route of Nrf2 activation (220). Other experimental work suggests that flavonoid based combinations may promote p62 mediated Keap1 sequestration and prolong Nrf2 signaling after dimethyl fumarate exposure (221). These findings support a mechanistic hypothesis for enhanced antioxidant defense, but they remain mainly preclinical and should not be presented as confirmed clinical benefit in MS.

Timing and formulation may also influence combination effects, but these areas remain early in development. Sequential dosing strategies, in which flavonoids are administered before DMTs, have been proposed to prime interferon related signaling and improve downstream immunoregulatory responses (214). Fixed dose nanoformulations combining flavonoids with DMTs have also been reported to improve pharmacokinetic and pharmacodynamic control and treatment adherence in experimental or early translational settings (222). However, these findings should be interpreted cautiously because improved delivery or adherence does not necessarily prove improved MS disease control. Such approaches require validation in well-designed MS trials before claims about superior efficacy, dose reduction or improved long term outcomes can be made.

The EGCG and glatiramer acetate combination further illustrates why synergy claims should be cautious. In relapsing EAE and related experimental models, EGCG combined with glatiramer acetate showed enhanced neuroprotective and anti-inflammatory effects compared with either treatment alone (71). However, these effects were not consistent across disease settings, as the same combination did not improve established chronic EAE and reduced some of the axonal and myelin preservation observed with EGCG alone (223). Human data also do not confirm clinical synergy. In relapsing remitting MS, adding EGCG to stable glatiramer acetate for 18 months was generally safe, but it did not improve the main MRI outcome compared with placebo (224). A smaller 12 week crossover study in glatiramer acetate treated patients reported changes in exercise energy metabolism after EGCG, but it was not designed to test relapse activity, disability progression, remyelination or MRI lesion control (225). Similarly, luteolin combined with interferon beta has shown additive suppression of inflammatory responses in immune cells from MS patients, but this remains an *in vitro* observation and does not establish clinical efficacy (226).

Overall, flavonoid and DMT combinations should be considered hypothesis generating rather than clinically validated. Future studies should include formal interaction testing, pharmacokinetic and pharmacodynamic endpoints, dose optimization, liver safety monitoring, assessment of CYP and transporter effects, and clinically meaningful outcomes such as relapse rate, MRI activity, disability progression, fatigue, cognition, brain atrophy and remyelination markers (216, 217, 222, 224, 225).

## 13 Comparative analysis: flavonoids versus conventional therapies in multiple sclerosis management

Comparative evidence suggests that flavonoid-containing adjuncts may supplement conventional DMT in MS by engaging neuroprotective and anti-inflammatory pathways that are only partially addressed by DMT (**Table 4**). Established DMTs diminish relapses and MRI activity by immunomodulation but are still burdened by side events and minimal impact on neurodegeneration (227, 228). Preclinical and translational study demonstrates that flavonoids such as epigallocatechin-3-gallate (EGCG), luteolin, and quercetin operate on NF-κB, Nrf2, microglia, mitochondria, and the gut–brain axis providing a mechanistic rationale for future adjunct studies with approved DMTs (191, 229).

**Table 4:** Comparative analysis: flavonoids versus conventional therapies in multiple sclerosis management.

Domain	Flavonoids	Conventional DMTs	Reference
Primary Mechanisms of Action	• Neuroprotective • Anti-inflammatory • Modulate NF-κB, Nrf2, microglia, mitochondrial pathways, gut–brain axis	• Immunomodulation • Reduce relapse rates and MRI activity	(191, 227–229)
Therapeutic Scope	Address oxidative stress, neurodegeneration, microglial activation mechanisms not fully targeted by DMTs	Effective in preventing inflammatory relapses but limited in slowing neurodegeneration	(227–229)
Combination evidence	• EGCG + GA: Enhanced neuroprotection, fewer infiltrates, increased axonal outgrowth in EAE and cellular models (230) • Luteolin + Interferon-β: Additive suppression of pro-inflammatory cytokines	• GA and Interferon-β well-established but show plateaued benefits when used alone	(71, 226)
Limitations of Synergy	• EGCG + GA ineffective in chronic EAE stages due to disease-stage and dosing constraints	• Long-term side effects (injection reactions, flu-like symptoms, hepatic strain) limit tolerability	(227, 228, 224, 225)
Clinical Trial Evidence	• EGCG + GA (18-month RCT): No significant increase in T2-lesion-free patients, but safe • EGCG crossover study: Improved exercise energy metabolism in GA-treated RRMS	• Strong evidence for relapse reduction and MRI activity control, but persistent neurodegeneration	(227, 228, 224, 225)
Safety Profile	Generally favourable; antioxidant and metabolic support may reduce DMT-related stress	Varies by agent; adverse-event burden may impact adherence and long-term use	(227, 224, 225)
Overall Contribution	Complements DMTs by reinforcing neuroprotective and anti-inflammatory pathways	Core disease-modifying effects via immunomodulation; incomplete neuroprotection	(71, 189, 227, 228, 223–226)

Potential interaction between EGCG and glatiramer acetate (GA) has been explored in preclinical and clinical settings, but confirmed clinical synergy has not been established. EGCG and GA has previously been reported in EAE and *in vitro* models, and the combination of EGCG and GA enhanced neuroprotection, reduced inflammatory infiltrates, and promoted axonal outgrowth beyond monotherapy (71). However, in chronic EAE, the same combination failed to outperform single agents, underscoring disease-stage and dosing contingencies (223). A randomized controlled trial in RRMS found that adding epigallocatechin gallate for 18 months to stable glatiramer acetate did not significantly increase the number of patients with no new T2 MRI lesions compared with placebo, but it was generally safe and well tolerated (224). In a separate 12-week crossover trial in GA-treated RRMS patients, EGCG improved exercise energy metabolism, a symptom-relevant effect that might complement DMT efficacy (225). Beyond EGCG, flavonoid-DMT interactions are an emerging area of investigation. In addition, *in vitro* combination of luteolin with interferon-β additively suppressed pro-inflammatory cytokine production peripheral blood mononuclear cells from MS patients, suggesting a potential augmentation of interferon therapy (226). Because most combination data are derived from EAE, *in vitro*, or small clinical studies not designed to prove synergy, the comparisons below should be interpreted as hypothesis generating rather than evidence of established clinical benefit.

## 14 Challenges and limitations in flavonoid research for multiple sclerosis

Despite promising preclinical findings, several pharmacokinetic and formulation barriers continue to limit the clinical translation of flavonoids in MS. Human studies show low and highly variable systemic exposure, with plasma metabolite concentrations often remaining in the low micromolar range, reflecting limited absorption, first pass metabolism and gut liver biotransformation (91, 231). Importantly, the compounds detected in human plasma are often not the same free aglycones used in many cell culture studies. Instead, flavonoids are commonly present as glucuronidated, sulphated, methylated or mixed conjugates, which may differ from the parent compound in activity, tissue distribution and target engagement (224, 231, 232). This creates a major gap between *in vitro* potency and clinically relevant dosing. BBB penetration is also uncertain, because CNS exposure appears to be compound and metabolite dependent and generally limited, making it difficult to confirm whether sufficient concentrations reach MS relevant targets in the brain and spinal cord (233).

Interindividual variability further complicates clinical translation. Glycoside form, food matrix, gut microbiota composition, intestinal transport, hepatic phase II metabolism and transporter activity can all influence systemic exposure. Flavonoids may also interact with disposition pathways and transporters such as OATPs and BCRP, which raises the possibility of food drug interactions and unpredictable exposure when used with approved MS DMTs (224, 232–234). Such interactions may alter either flavonoid exposure or DMT exposure and therefore should be assessed directly rather than assumed to be beneficial. Liver safety is also important, particularly for concentrated preparations such as high dose EGCG and for patients receiving DMTs that already require hepatic monitoring. Recent EGCG trials in relapsing remitting and progressive MS also show that mechanistic promise and oral dosing feasibility do not necessarily translate into clear MRI or clinical benefit (227, 228).

Strategies to improve CNS delivery are promising but remain mostly experimental. Liposomes, polymeric nanoparticles and related carrier systems may improve solubility, stability and tissue exposure, but they also introduce challenges related to manufacturing, long term stability, regulatory quality, cost and scalable production (235). Therefore, improved delivery systems should not be presented as having already solved the bioavailability or BBB penetration problem. Future studies should include standardized preparations, pharmacokinetic and pharmacodynamic endpoints, metabolite profiling, CNS exposure assessment when feasible, liver safety monitoring, CYP and transporter interaction testing, and careful assessment of interactions with approved DMTs. Because unsupervised botanical use is common among patients and interaction profiles remain uncertain, flavonoid use in MS should be clinician guided until stronger clinical and safety evidence is available (236).

## 15 Future perspectives and emerging opportunities in flavonoid research for multiple sclerosis

Advances in next generation single-cell and spatial omics now allow cell type–resolved readouts of pathology and treatment response across MS lesion ecosystems. Single nucleus RNAseq has revealed disease associated oligodendroglia and stratified patients by distinct white matter glial programs, creating a framework to detect pharmacodynamic signatures of flavonoids at cellular resolution (237, 238). Spatial transcriptomics and single-cell spatial maps can further link microenvironmental niches (reactive rims, remyelinating cores, perivascular spaces) to flavonoid modulated pathways e.g., oxidative stress, inflammasome activity, and phagocytic remodeling supporting rational combination with DMTs and stage specific deployment (239, 240).

### 15.1 Proteomic innovations for target discovery

High resolution quantitative proteomics (nano LC-MS) has matured into a discovery engine for mechanism of action and target deconvolution. Data independent acquisition (DIA) expands coverage and quantitation, while multiplexed workflows enable time and dose resolved profiling of flavonoid exposures in patient derived cells or CSF (241–244). Phospho proteomics adds network level insight into kinase signaling perturbed by flavonoids (e.g., NF κB, MAPK, Nrf2) and can be analyzed with dedicated toolchains (PhosR) to infer upstream kinases and pathway nodes for pairing with DMTs (230). Together, these platforms support unbiased identification of actionable protein complexes, PTMs, and signaling hubs that mediate flavonoid responses in MS.

### 15.2 Nanotechnology for targeted delivery

State of the art nanocarriers address poor oral bioavailability and BBB penetration by leveraging receptor mediated transcytosis and stimuli responsive release. For flavonoids, solid lipid or nanostructured lipid carriers functionalized with transferrin or RVG29 have improved brain delivery and neuronal uptake in preclinical models; EGCG or quercetin loaded formulations illustrate feasibility (245–247). Broad reviews of BBB targeted nanomedicines highlight lactoferrin/transferrin decorated systems, peptide ligands, and pH responsive polymers as adaptable scaffolds for MS relevant neuroinflammation (245, 248). Translational priorities include scale up, long term stability, and lesion specific targeting in demyelinating contexts.

### 15.3 AI driven discovery and personalized design

AI is accelerating natural product drug discovery via genome/metabolome mining, target prediction, *de novo* design, and multi omics integration; these capabilities are directly applicable to flavonoid scaffolds (249). For combinations, open platforms (e.g., SynergyFinder) and deep learning models (transformers, graph neural networks) enable in silico prioritization of DMT-flavonoid pairs and responder stratification using transcriptomic or proteomic features (250–252). In parallel, AI guided medicinal chemistry can optimize bioavailability and BBB properties while preserving polypharmacology (253). Embedding these models within MS specific datasets (single cell, imaging, clinical phenotypes) should yield patient tailored flavonoid regimens.

### 15.4 Epigenetic and microbiome interactions

Flavonoids modulate epigenetic regulators (DNMTs, HDACs) and immune inflammatory transcriptional programs. EGCG and quercetin exemplify DNMT inhibition, histone acetylation changes, and miRNA modulation relevant to neuroinflammation (254–256). Concurrently, MS associated dysbiosis (e.g., increased *Akkermansia*/*Methanobrevibacter*) and functional transfer of MS microbiota that exacerbates CNS autoimmunity in mice position the gut-brain axis as a co target for flavonoid intervention (257–259). Combining epigenetic readouts with metagenomic or metabolomic profiling could identify microbial consortia that bioactivate flavonoids into efficacious metabolites and predict responders.

### 15.5 Gene editing and synthetic biology solutions

Engineering microbial cell factories (e.g. yeast, *E. coli*) enables scalable production and late stage diversification of flavonoids with improved stability and BBB permeability (260–262). Beyond manufacturing, engineered live biotherapeutics can be programmed to sense intestinal cues and deliver therapeutics *in situ*, an approach increasingly feasible for modulating flavonoid pathways and the MS relevant microbiome (263, 264). Recent toolkits in *E. coli* Nissle expand chassis control for payload production and context aware release, offering a route to personalized, locally delivered flavonoid therapy that circumvents first pass metabolism (265).

### 15.6 Systems biology and network medicine approaches

Network centric analyses integrating multi omics with imaging and clinical phenotypes have revealed multiscale MS modules that track tissue damage and severity (266). Applying these frameworks to flavonoid perturbation experiments can identify convergence nodes (e.g., oxidative stress, microglial activation) and nominate rational DMT partners. Standardized workflows and software (e.g., Cytoscape) support reproducible mapping of flavonoid affected interactomes, while trends in multi tissue systems biology guide data integration and translation to biomarkers (267, 268).

### 15.7 Future perspectives on organ on a chip in flavonoid based MS therapy

Human BBB and neurovascular unit on chip systems recapitulate barrier function, immune trafficking, and inflammatory endothelial signaling, enabling rigorous tests of flavonoid transport, metabolism, and efficacy under flow (269, 270). iPSC derived, patient specific BBB chips capture inter individual variability and disease specific defects, supporting precision dose selection and interaction testing with DMTs (271). Next-generation NVU chips mimicking rim-active lesions and T-cell transmigration could allow quantification of the degree to which flavonoid-loaded nanocarriers or free flavonoids restore barrier integrity or modulate glial responses, enabling preclinical-to-clinical translation.

## 16 Balanced interpretation of clinical evidence and limits of translation

Flavonoids are still biologically plausible adjunctive candidates for MS, but the existing data does not justify presenting them as established disease-modifying therapies. The most powerful beneficial effects remain limited to experimental systems and have not been reliably transferred to human MRI or disability outcomes (224, 272). Of the compounds reviewed, EGCG has the most direct evidence in the clinic for MS patients. In a multicenter, randomized, double blind phase II trial of patients with relapsing remitting MS on stable glatiramer acetate, oral EGCG up to 800 mg daily for 18 months was safe but was not superior to placebo for new T2 weighted lesions, lesion volume, contrast enhancing lesions, brain volume change, Expanded Disability Status Scale (EDSS), MS Functional Composite (MSFC), or annualized relapse rate (224). In a randomized, double blind phase II trial in progressive MS, EGCG up to 1200 mg daily for 36 months did not improve brain atrophy, radiological outcomes or clinical disease markers, and one participant withdrew from treatment due to increased aminotransferases, highlighting the need for liver safety monitoring at high supplemental doses (92). Smaller studies suggest possible physiological effects but the results should not be over interpreted. For example, in a small crossover trial of patients with relapsing remitting MS treated with glatiramer acetate, EGCG 600 mg daily for 12 weeks altered some measures of energy metabolism, but the study was not designed to assess relapse activity, disability progression, remyelination or MRI lesion control (225). Also, there is no clear positive evidence from flavonoid-rich botanical preparations. A randomized placebo controlled trial of 120 mg twice daily for 12 weeks of Ginkgo biloba extract containing flavonol glycosides did not improve cognitive performance, suggesting that mechanistic rationale does not necessarily translate into clinically meaningful neurological benefit (93). Efficacy in preclinical models should be viewed in the context of the limitations of the models. EAE is useful as a model for immune mediated CNS inflammation, in particular CD4 T cell mediated autoimmunity but human MS is a more complex disease that also involves CD8 T cells, B cells, compartmentalized CNS inflammation, cortical lesions, chronic active lesions and progressive neurodegeneration (272, 273). The cuprizone model is a useful model to study oligodendrocyte injury, demyelination and remyelination, however, because of its toxin driven nature with limited participation of peripheral adaptive immune cells, positive results in this model are mostly supportive of the effects on glial injury and repair biology, rather than systemic MS immunotherapy (94, 274). Glatiramer acetate + EGCG also shows the gulf between promising preclinical results and uncertain clinical translation. This combination had coactive neuroprotective and anti-inflammatory effects in a relapsing EAE model but did not improve established chronic EAE and even reduced the axonal and myelin preservation observed with EGCG alone (275, 276). Dosing and formulation approaches vary, leading to further interpretation challenges, including preventive dosing, treatment after disease onset, oral gavage, purified compounds, botanical extracts, nanoformulations, and human regimens (ranging from EGCG 600 mg daily for 12 weeks to 1200 mg daily for 36 months) (93, 224, 225). A further important barrier is pharmacokinetic variability, because the human exposure to quercetin depends on the glycoside form, plant matrix, absorption and phase II metabolism and the circulating forms after dietary intake are mainly methylated, glucuronidated, sulphated or mixed conjugates and not the free aglycone often used in cell culture experiments (277). Therefore, future studies should incorporate pharmacokinetic and pharmacodynamic endpoints, metabolite profiling, monitoring of liver safety, standardized preparations, sex and disease phenotype stratification, assessment of interaction with approved DMTs and clinically meaningful outcomes such as relapse activity, MRI lesions, disability progression, cognition, fatigue, brain atrophy and remyelination markers before being able to claim that flavonoids modify MS progression.

## 17 Conclusion

Current evidence suggests that selected flavonoids can modulate inflammatory, oxidative stress, glial, mitochondrial, and repair related pathways relevant to MS. However, most supportive data remain preclinical or mechanistic, and human evidence is limited and mixed. EGCG has the most direct clinical evidence among the flavonoids discussed, but randomized studies have not shown consistent benefit on core MRI, relapse, disability, or brain atrophy outcomes. Therefore, flavonoids should be viewed as biologically plausible adjunct candidates requiring clinical validation, not as established disease modifying or remyelinating therapies. Future research should focus on standardized formulations, clinically relevant dosing, pharmacokinetic and pharmacodynamic endpoints, metabolite profiling, liver safety monitoring, interaction assessment with approved DMTs, and clinically meaningful outcomes such as relapse activity, MRI lesions, disability progression, cognition, fatigue, brain atrophy, and remyelination markers. Advances in delivery technologies, systems biology, artificial intelligence, and synthetic biology may help prioritize candidate compounds and improve study design, but these approaches should be considered enabling tools rather than proof of clinical efficacy.
